# Supplementary material for: WS2 moiré superlattices derived from mechanical flexibility for hydrogen evolution reaction
Source: Nat Commun. 2021 Aug 20;12:5070. doi: 10.1038/s41467-021-25381-1 (PMC8379161; doi:10.1038/s41467-021-25381-1)
Supplement: Supplementary file 1 — Supplementary Information [file 41467_2021_25381_MOESM1_ESM.pdf]

Supplementary Information

WS_2 moiré superlattices derived from mechanical flexibility for hydrogen evolution reaction

Lingbin Xie,[a] Longlu Wang,[b,*] Weiwei Zhao,[a] Shujuan Liu,[a] Wei Huang,[a,c,*] and Qiang Zhao[a,b,*]

[a] State Key Laboratory of Organic Electronics and Information Displays & Jiangsu Key Laboratory for Biosensors, Institute of Advanced Materials (IAM) & Institute of Flexible Electronics (Future Technology), Nanjing University of Posts & Telecommunications, 9 Wenyuan Road, Nanjing 210023, China

[b] College of Electronic and Optical Engineering & College of Microelectronics, Jiangsu Province Engineering Research Center for Fabrication and Application of Special Optical Fiber Materials and Devices, Nanjing University of Posts & Telecommunications (NUPT), 9 Wenyuan Road, Nanjing 210023, China

[c] Frontiers Science Center for Flexible Electronics (FSCFE), MIIT Key Laboratory of Flexible Electronics (KLoFE), Northwestern Polytechnical University, Xi'an 710072, Shaanxi, China

E-mail addresses: wanglonglu@njupt.edu.cn (L. Wang), provost@nwpu.edu.cn (W. Huang), iamqzhao@njupt.edu.cn (Q. Zhao)

Supplementary Figures

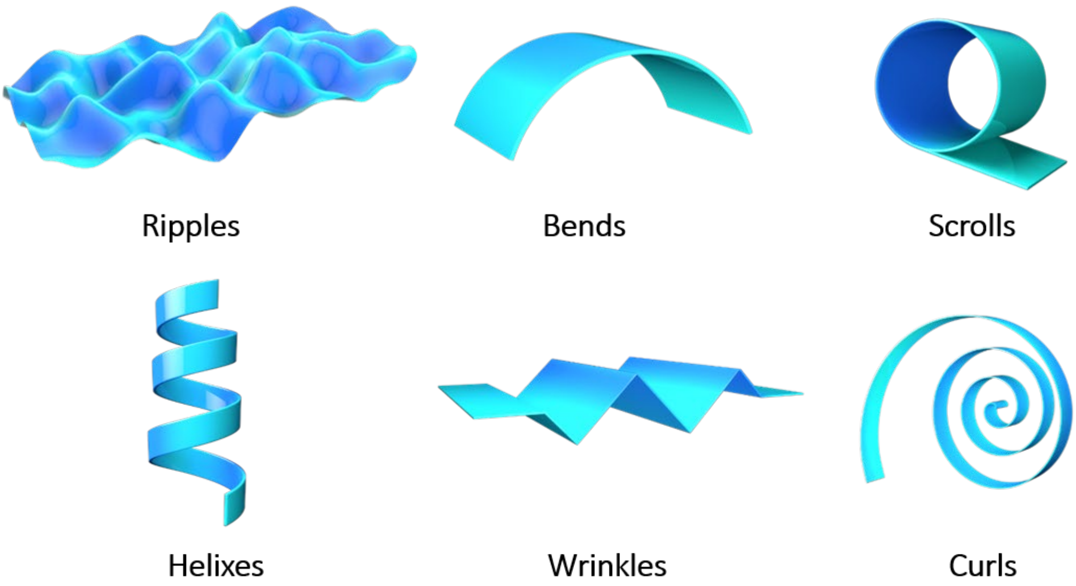

Supplementary Figure 1 | Different deformation states of two-dimensional materials.

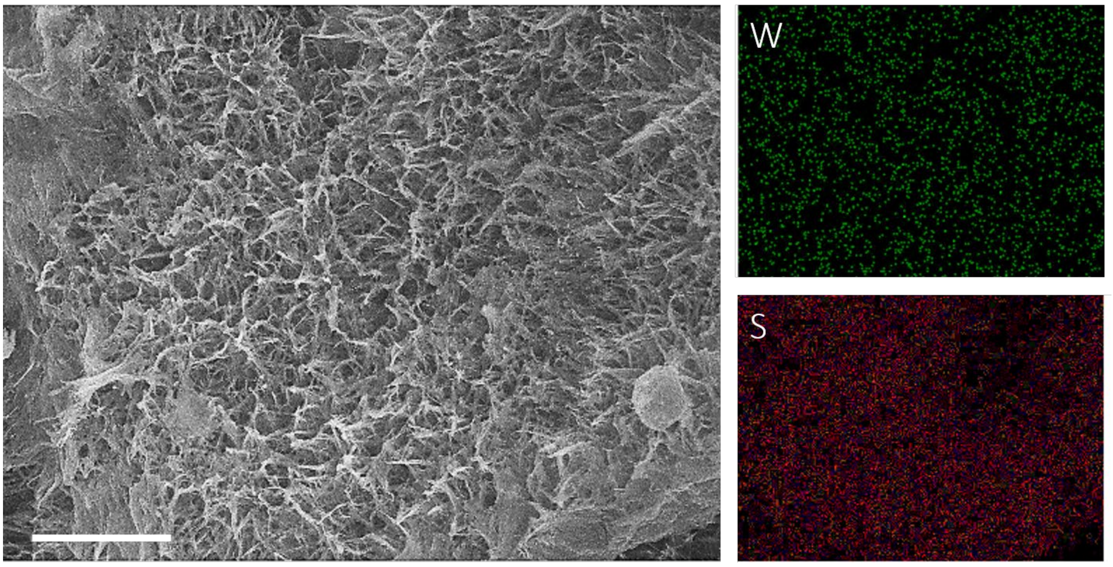

Supplementary Figure 2 | SEM and the corresponding EDS element mapping images of WS_2 nanoarrays. Scale bar, 10 μm.

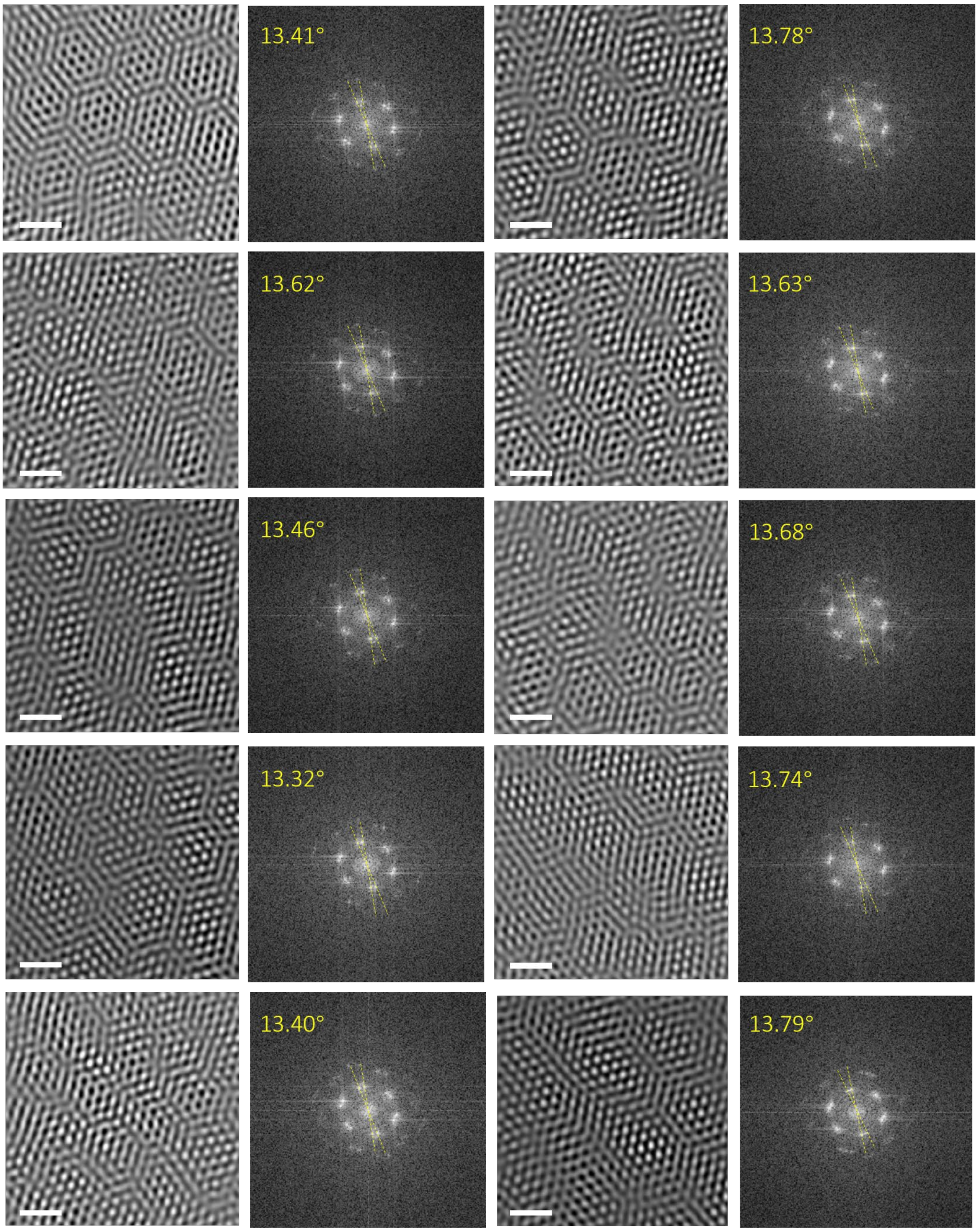

Supplementary Figure 3 | Examples of Moiré pattern using HRTEM images randomly selected from different WS_2 nanocones and their corresponding FFT images. Scale bar, 1 nm.

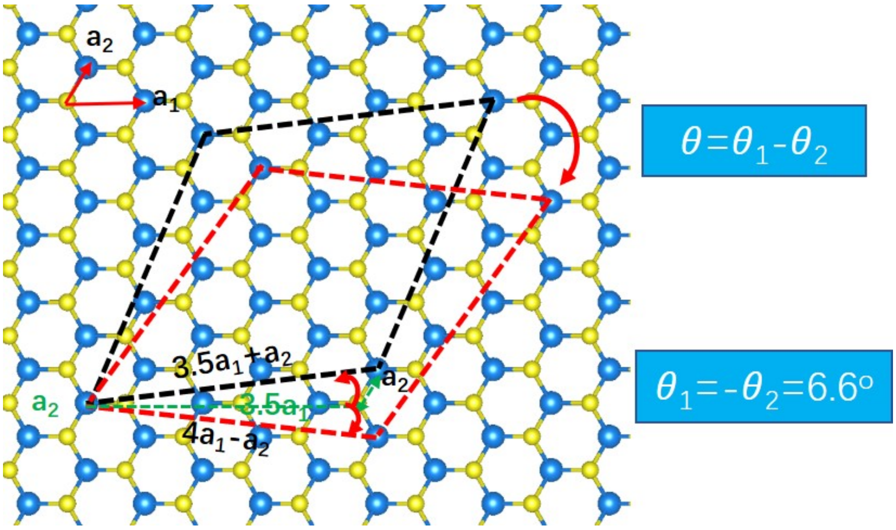

Supplementary Figure 4 | Schematic plot of the skewed supercell in a hexagonal lattice. The skewed angle θ_1 or θ_2 is defined as the angle between the basis vector (n$\mathbf{a_1}$ + m$\mathbf{a_2}$) and the zigzag direction. A pair of such skewed supercell with either exact or close matching of their lateral periodicity can become commensurate by rotating one relative to the other. The formed twisted bilayer sheet has a rotation angle of $\theta = \theta_1 - \theta_2$.

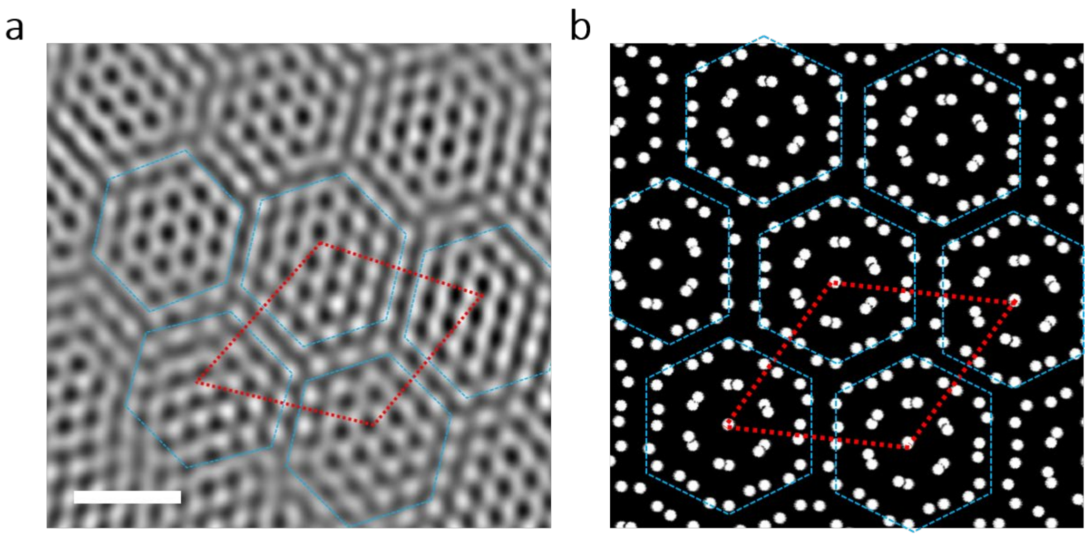

Supplementary Figure 5 | (a) HRTEM image of WS_2 MSLs. Scale bar, 1 nm. **(b)** Simulated HRTEM image of WS_2 MSLs.

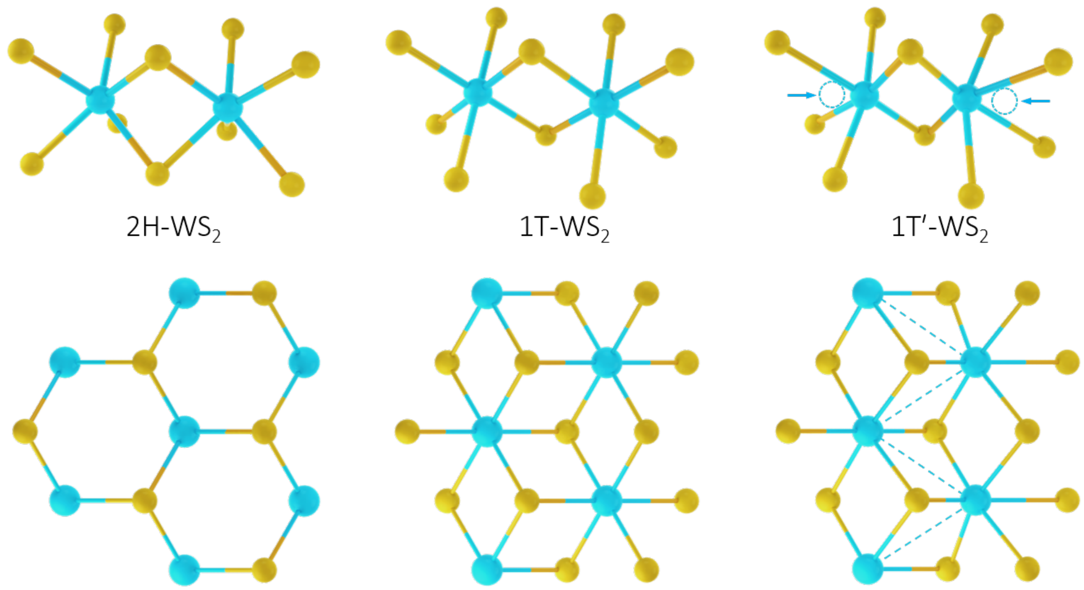

Supplementary Figure 6 | Top view and side view of atomic structure model of WS_2 structural polymorphs. Yellow and cyan balls represent S and W atoms, respectively.

a b

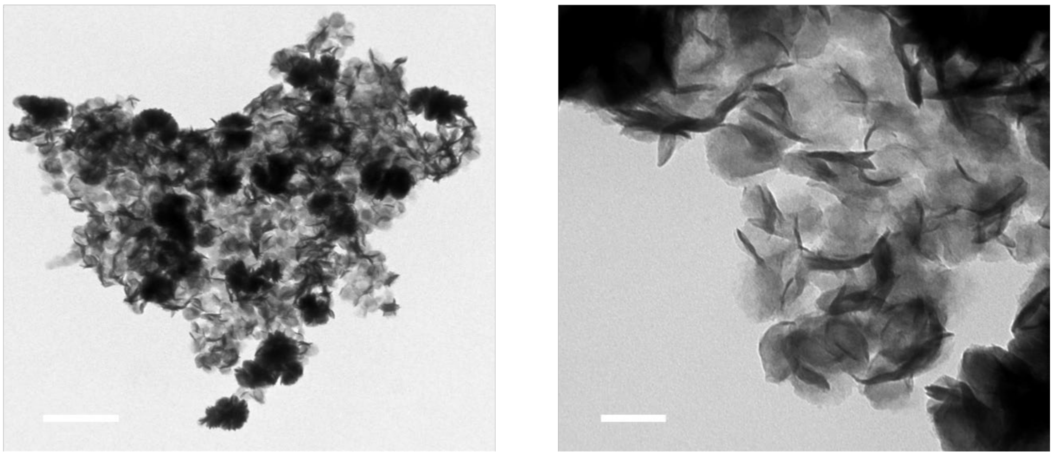

Supplementary Figure 7 | (a, b) TEM images of 1T'-WS_2 NSs. Scale bar, 500 nm (a) and Scale bar, 100 nm (b).

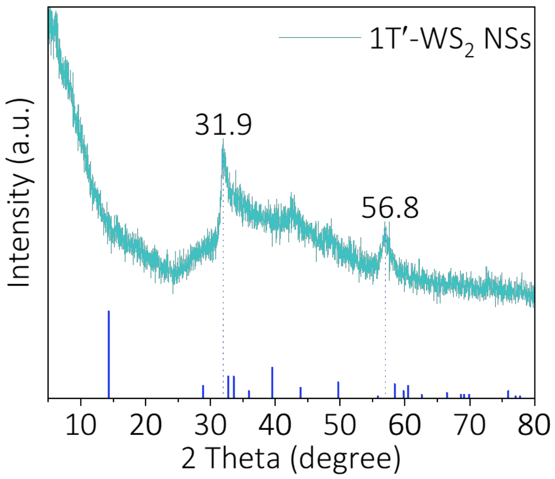

Supplementary Figure 8 | XRD patterns of 1T'-WS_2 NSs. Using PDF#08-0237 for reference.

The XRD patterns of WS_2 samples are presented in **Supplementary Fig. 8**, exhibit well resolved diffraction peaks. The XRD peaks in regions of 31–32° and 56–59° can be used to identify 1T′ or 2H WS_2. As shown in Figure S4, the XRD peaks of 1T′-WS_2 at 31.9° and 56.8° are attributed to the (040) and (440) planes of the 1T′ phase.

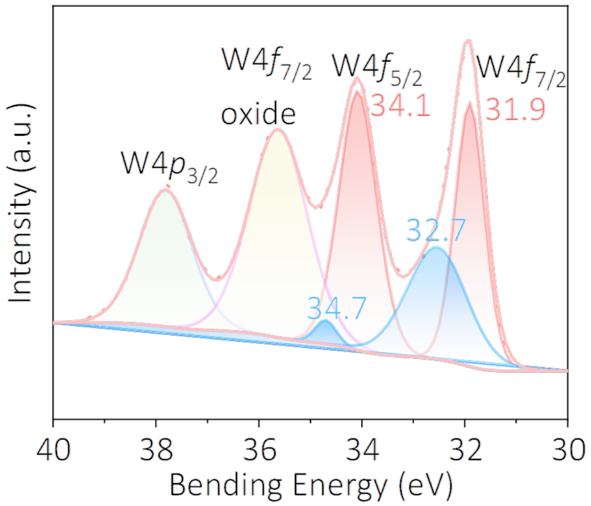

Supplementary Figure 9 | XPS spectra of W 4f binding energy of 1T'-WS_2 NSs.

The core level peaks of W4$f_{7/2}$ and W4$f_{5/2}$ in XPS spectra is an efficient way to distinguish the metallic and 2H phase. As shown in **Supplementary Fig. 9**, double peaks located at 31.9 eV and 34.1 eV are ascribed to the core levels of W4$f_{7/2}$ and W4$f_{5/2}$ of 1T′-WS_2, respectively. Two strong peaks of 1T'-WS_2 NSs at 32.7 eV (W4$f_{7/2}$) and 34.7 eV (W4$f_{5/2}$) are the characteristics of W for 2H-WS_2. The results indicate the formation of 1T'-WS_2.

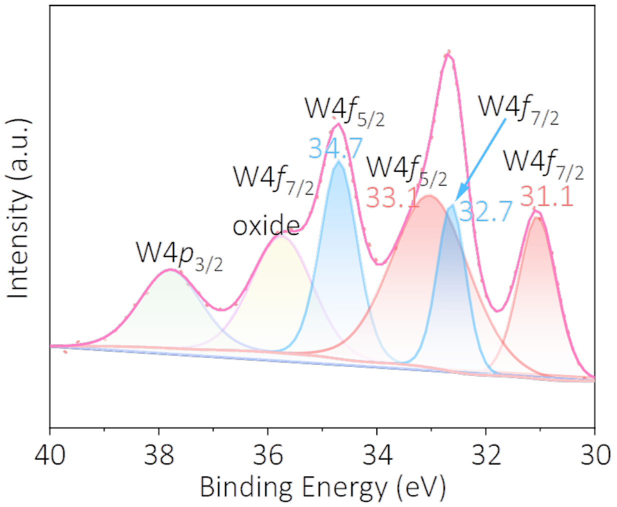

Supplementary Figure 10 | XPS spectra of W signals recorded from WS_2 MSLs.

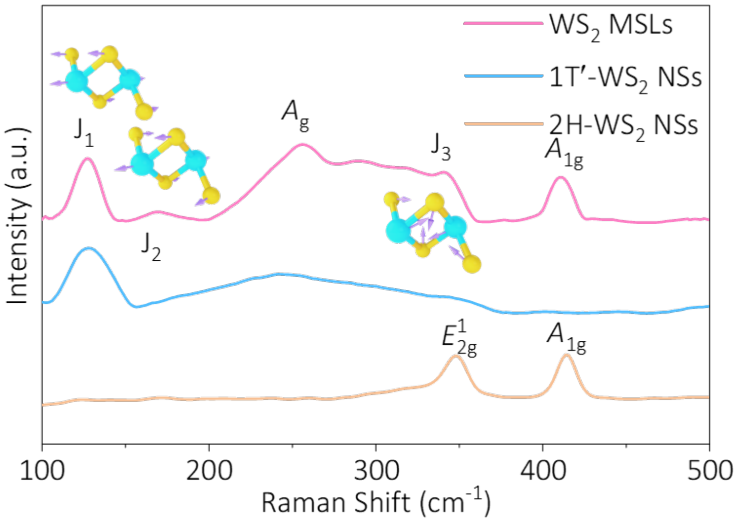

Supplementary Figure 11 | Raman spectroscopy of as-obtained WS_2 MSLs, 1T′-WS_2 NSs and 2H-WS_2 NSs samples, respectively. Yellow and cyan balls represent S and W atoms, respectively.

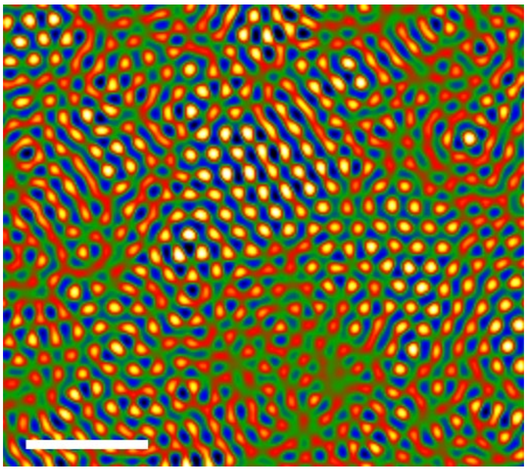

Supplementary Figure 12 | HRTEM images of in-plane 1T′@2H-WS_2 heterostructures, showing the crystalline fringe spacing of 1T′ and 2H phases, Scale bar, 5 nm.

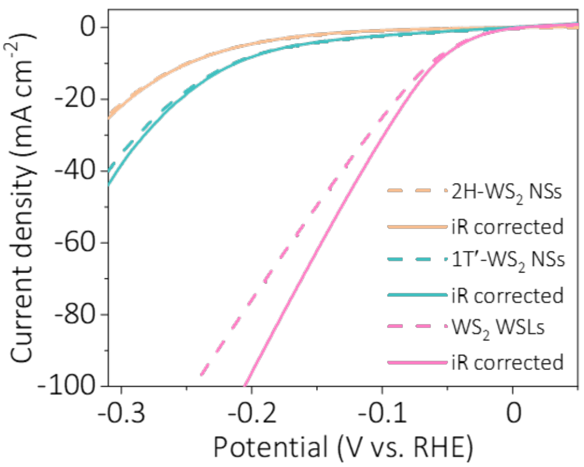

Supplementary Figure 13 | Polarization curves of the different WS_2-based catalysts. All the samples were tested in Ar-bubbled 0.5 M H_2SO_4 solutions. Curves both with and without iR corrections are provided for each sample.

Correcting for Ohmic losses throughout the system, including solution resistance, allows us to better compare the true catalytic activity of our WS_2 MSLs to other WS_2-based catalysts. Using the series resistance (R_s) determined from electrochemical impedance spectroscopy experiments, we correct our polarization measurements for these iR losses (**Fig. 3a**). The results show that WS_2 MSLs achieves 10 mA cm^{-2} at potentials as low as −60 mV vs. RHE and the corresponding Tafel slope is reduced to 40 mV decade^{-1} (**Fig. 3b**), making WS_2 MSLs one of the most catalytic WS_2 materials reported.

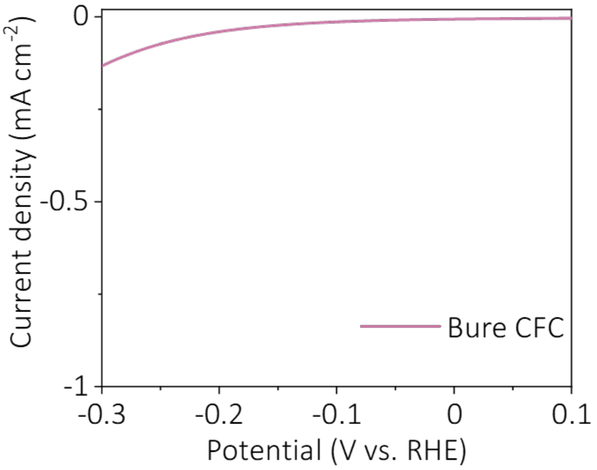

Supplementary Figure 14 | The polarization curve of bare CFC (1 cm^2), indicating that the bare CFC exhibits negligible electrocatalytic performance for the HER.

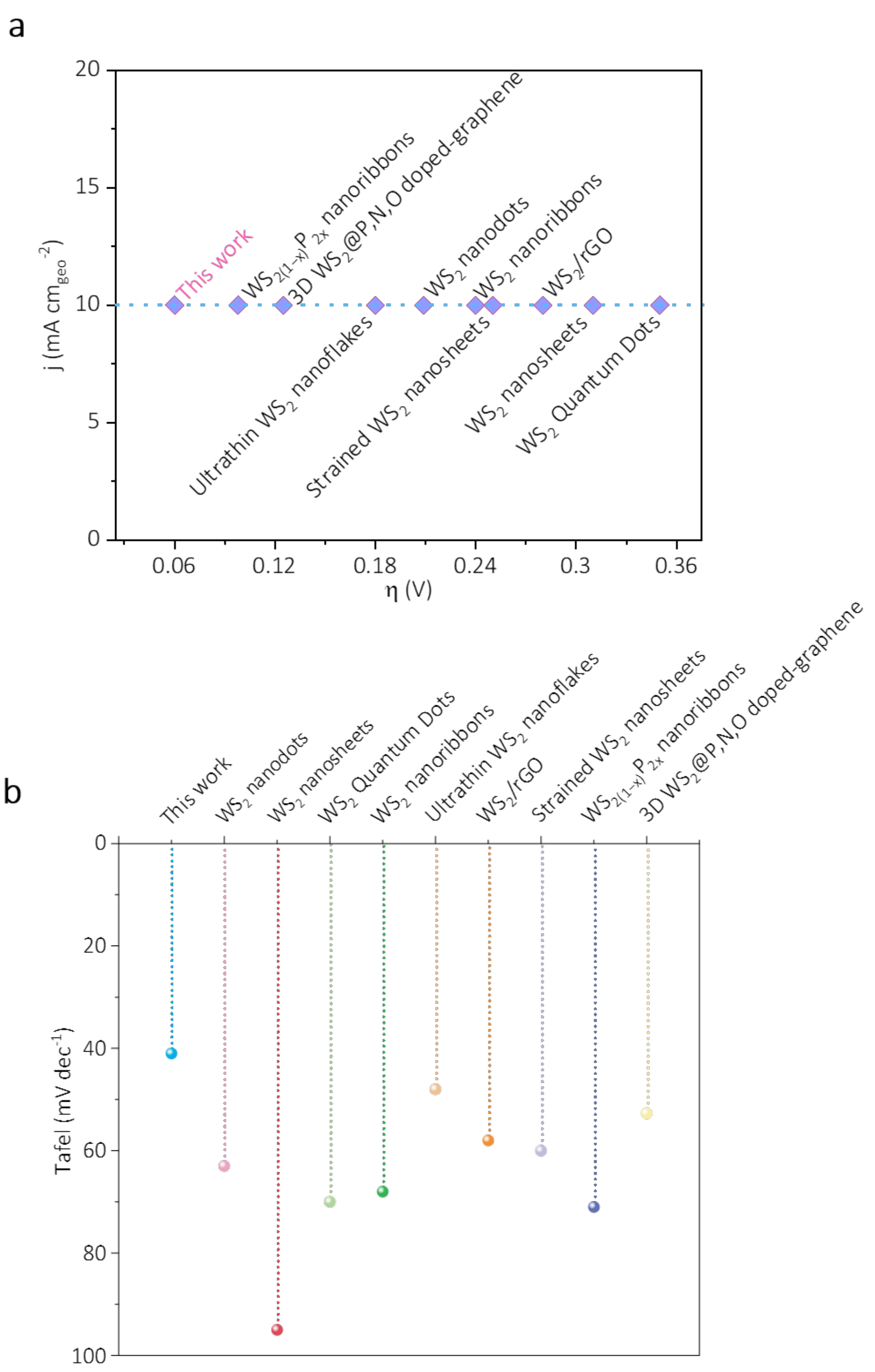

Supplementary Figure 15 | (a) Comparison of overpotential required to generate a current density of 10 mA cm^{-2} on various WS_2-based catalysts, including WS_2 nanodots (Adv. Mater. **2018**, 30, 1705509), $WS_{2(1-x)}P_{2x}$ nanoribbons (Small **2017**, 13, 1603706),

3D WS_2@P, N, O doped-graphene nanocomposites (Adv. Mater. **2015**, 27, 4234), WS_2 Quantum Dots (Adv. Funct. Mater. **2015**, 25, 1127), WS_2 nanoribbons (Adv. Energy Mater. **2014**, 4, 1301875), Ultrathin WS_2 nanoflakes (Angew. Chem., Int. Ed. **2014**, 53, 7860), WS_2/RGO nanocomposites (Angew. Chem., Int. Ed. **2013**, 52, 13751), Strained WS_2 nanosheets (Nat. Mater. **2013**, 12, 850). (**b**) The corresponding Tafel curves for various WS_2 based electrocatalysts derived from (a).

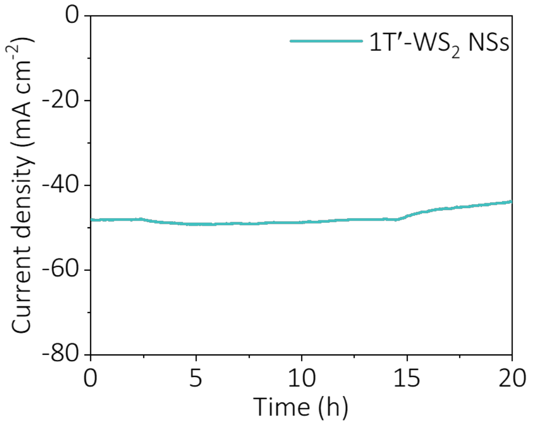

Supplementary Figure 16 | Continuous HER recorded from 1T′-WS_2 NSs coated CFC as working electrodes at a static overpotential of −0.35 V vs. RHE.

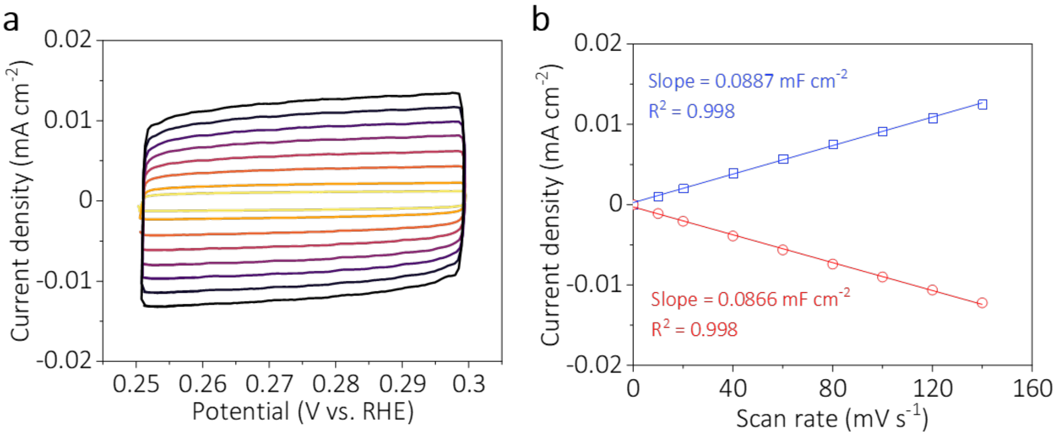

Supplementary Figure 17 | Double-layer capacitance measurements for determining electrochemically-active surface area for the substrate material (bare CFC) from voltammetry in Ar-bubbled 0.5 M H_2SO_4. (**a**) Cyclic voltammograms were measured in a non-Faradaic region of the voltammogram at the following scan rate: 10, 20, 40, 60, 80, 100, 120, and 140 mV s^{-1}. The working electrode was held at each potential vertex for 10 s before the beginning the next sweep. All current is assumed to be due to capacitive charging. (**b**) The cathodic (○) and anodic (□) charging currents measured at 0.275 V vs. RHE plotted as a function of scan rate.

The determined double-layer capacitance of the system is taken as the average of the absolute value of the slope of the linear fits to the data—here it is 0.0876 mF cm^{-2}.

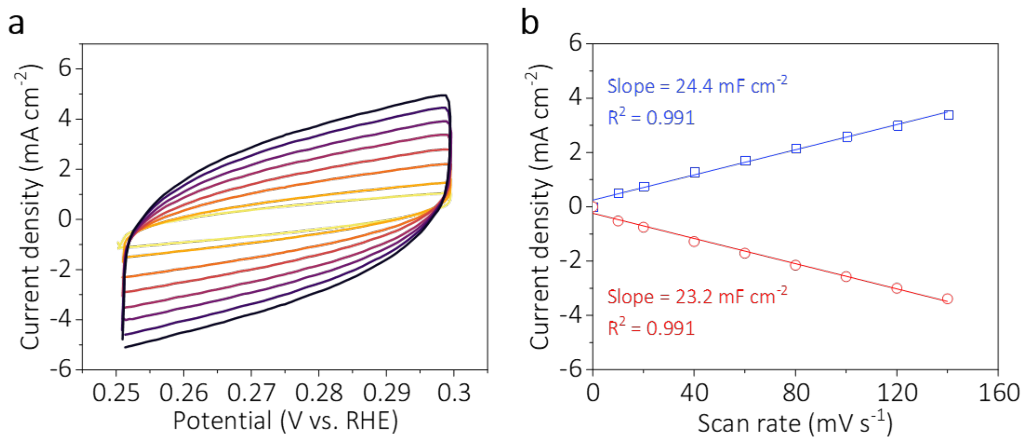

Supplementary Figure 18 | Double-layer capacitance measurements for determining electrochemically-active surface area for WS_2 MSLs sample from voltammetry in Ar-bubbled 0.5 M H_2SO_4. (**a**) Cyclic voltammograms were measured in a non-Faradaic region of the voltammogram at the following scan rate: 10, 20, 40, 60, 80, 100, 120, and 140 mV s^{-1}. The working electrode was held at each potential vertex for 10 s before the beginning the next sweep. All current is assumed to be due to capacitive charging. (**b**) The cathodic (○) and anodic (□) charging currents measured at 0.275 V vs. RHE plotted as a function of scan rate.

The determined double-layer capacitance of the system is taken as the average of the absolute value of the slope of the linear fits to the data—here it is 23.8 mF cm^{-2}. Assuming a general specific capacitance of 60 mF cm^{-2} gives an electrochemically active surface area of 396.6 cm^2_{ECSA} from this measurement.

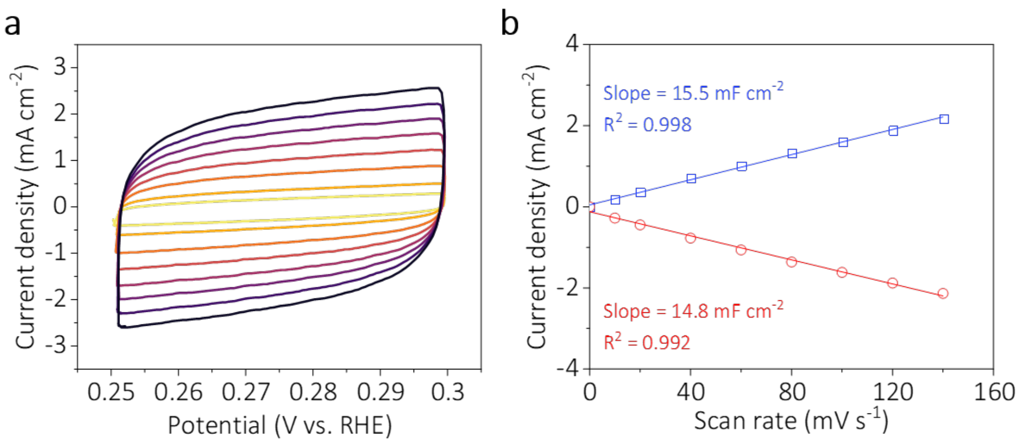

Supplementary Figure 19 | Double-layer capacitance measurements for determining electrochemically-active surface area for 1T'-WS_2 NSs sample from voltammetry in Ar-bubbled 0.5 M H_2SO_4. (**a**) Cyclic voltammograms were measured in a non-Faradaic region of the voltammogram at the following scan rate: 10, 20, 40, 60, 80, 100, 120, and 140 mV s^{-1}. The working electrode was held at each potential vertex for 10 s before the beginning the next sweep. All current is assumed to be due to capacitive charging. (**b**) The cathodic (○) and anodic (□) charging currents measured at 0.275 V vs. RHE plotted as a function of scan rate.

The determined double-layer capacitance of the system is taken as the average of the absolute value of the slope of the linear fits to the data—here it is 15.2 mF cm^{-2}. Assuming a general specific capacitance of 60 mF cm^{-2} gives an electrochemically active surface area of 253.3 cm^2_{ECSA} from this measurement.

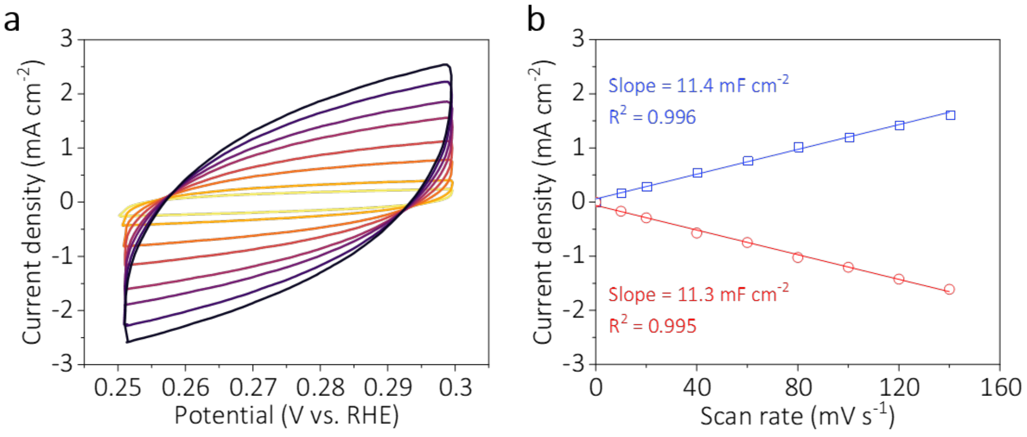

Supplementary Figure 20 | Double-layer capacitance measurements for determining electrochemically-active surface area for 2H-WS_2 NSs sample from voltammetry in Ar-bubbled 0.5 M H_2SO_4. (a) Cyclic voltammograms were measured in a non-Faradaic region of the voltammogram at the following scan rate: 10, 20, 40, 60, 80, 100, 120, and 140 mV s^{-1}. The working electrode was held at each potential vertex for 10 s before the beginning the next sweep. All current is assumed to be due to capacitive charging. (b) The cathodic (○) and anodic (□) charging currents measured at 0.275 V vs. RHE plotted as a function of scan rate.

The determined double-layer capacitance of the system is taken as the average of the absolute value of the slope of the linear fits to the data—here it is 11.4 mF cm^{-2}. Assuming a general specific capacitance of 60 mF cm^{-2} gives an electrochemically active surface area of 190.0 cm^2_{ECSA} from this measurement.

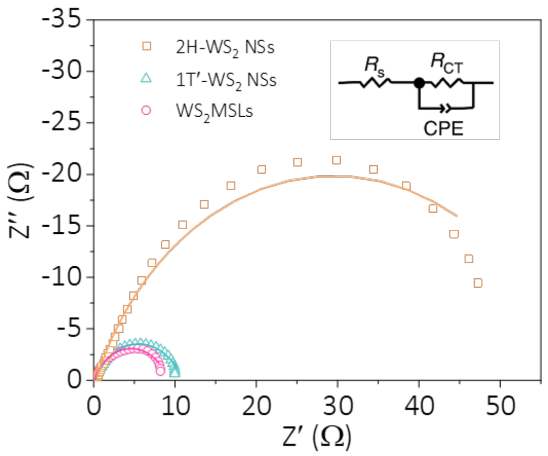

Supplementary Figure 21 | Nyquist plots for EIS measurements of WS_2 MSLs, 1T'-WS_2 NSs and 2H-WS_2 NSs, using the frequency in the range from 100 kHz to 0.1 Hz at 0.25 V (vs. RHE). The inset is the equivalent circuit model that contains the electrolyte resistance (R_s), constant phase element (CPE) and charge-transfer resistance (R_{ct}). *Z*' is the real impendence and Z" is the imaginary impedance.

Electrochemical impedance spectroscopy (EIS) measurements of the samples were performed using a 100 kHz–0.1 Hz frequency range and an amplitude of 10 mV at 0.25 V (vs. RHE). In the high frequency limit and under non-Faradaic conditions, the electrochemical system is approximated by the modified Randles circuit shown in the inset, where R_s denotes the solution resistance, CPE is a constant-phase element related to the double-layer capacitance, and R_{ct} is the charge-transfer resistance from any residual Faradaic processes. A semicircle in the low-frequency region of the Nyquist plots represents the charge transfer process, with the diameter of the semicircle reflecting the charge-transfer resistance. The real (Z') and negative imaginary (-Z") components of the impedance are plotted on the *x* and *y* axis, respectively. The fitting parameters are listed in **Supplementary Table 4**.

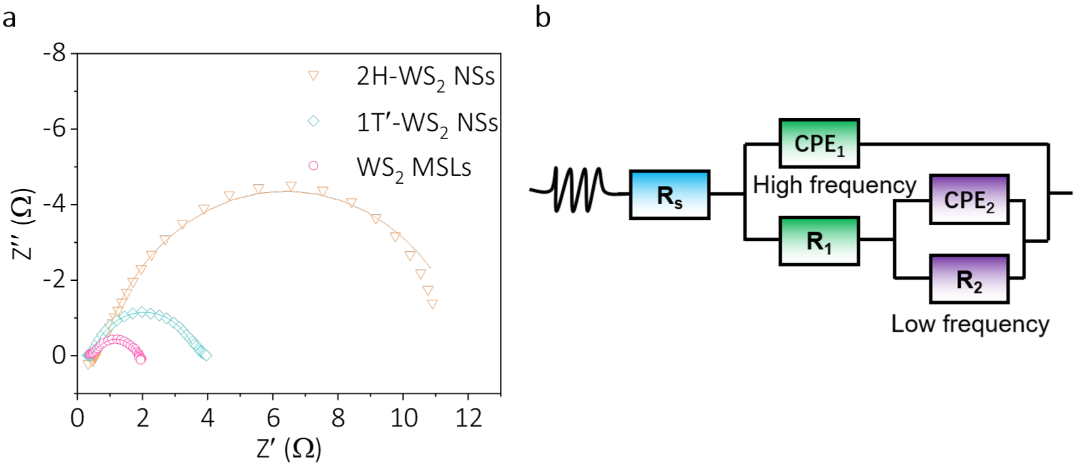

Supplementary Figure 22 | (a) Nyquist plots of impedance data for different WS_2 samples. (**b**) The equivalent circuit used to fit the data.

According to the polarization curve of the catalysts (**Figure 3a**), the EIS measurements of the samples were performed using a 100 kHz – 0.1 Hz frequency range and an amplitude of 10 mV at –50 mV (vs. RHE). **Supplementary Figure 22** shows Nyquist plots for impedance data of different WS_2 samples.

The experimental impedance data were fitted to the equivalent circuit model[1] by ZView software. The EIS data at a potential of −50 mV vs. RHE together with the corresponding fitted curve are displayed in **Supplementary Figure 22a** and the equivalent circuit model is seen to the EIS data very well. In addition, it is noted that equivalent circuits drawn for same electrochemical impedance spectroscopy are not unique.

In the two time constant parallel model the R_S resistance element is attributed to the uncompensated solution resistance, the high frequency time constant (τ_1, CPE_1-R_1) is related to the Faradaic resistance for the charge transfer process, R_{ct}, and double layer capacitance, C_{dl}, while the low frequency time constant (τ_2, CPE_2-R_2) is related to hydrogen adsorption, R_p and C_p.

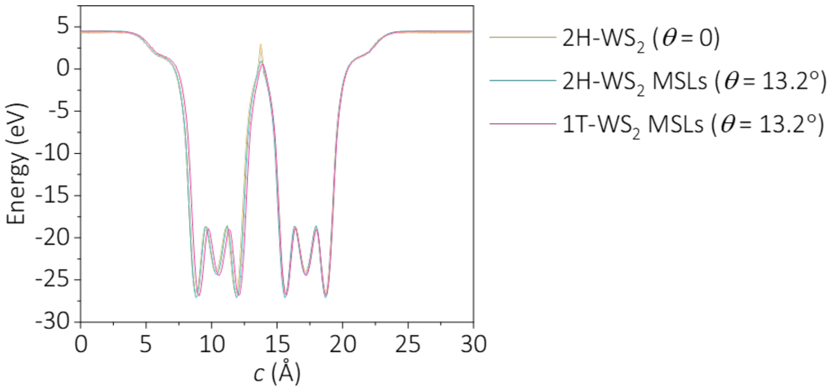

Supplementary Figure 23 | Total potentials for the superlattice with $\theta = 0$ and 13.2°.

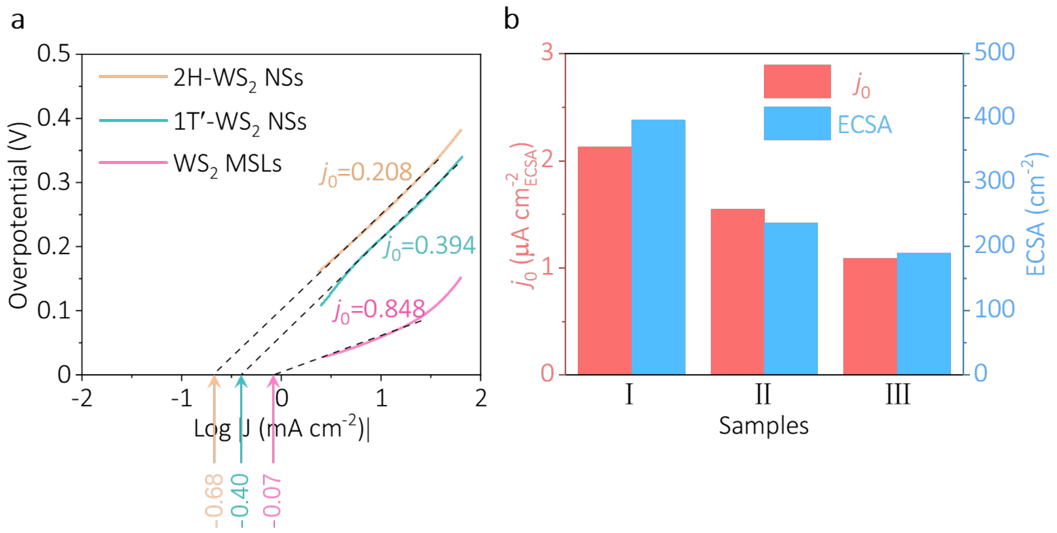

Supplementary Figure 24 | (a) Exchange current density calculation. Calculated exchange current for different studied WS_2 samples by using extrapolation methods (determined as the extrapolated x intercept for $\eta = 0$). (b) Comparison of the ECSA and the ECSA-normalized j_0 of (I) WS_2 MSLs, (II) 1T'-WS_2 NSs, (III) 2H-WS_2 NSs.

The exchange current densities (j_0) of the different samples were determined from the Tafel plots. The exchange current density was normalized by electrochemically active surface area (ECSA). The j_0 of 2.13 μA cm^{-2}_{ECSA} for WS_2 MSLs sample surpasses the values of 1.55 μA cm^{-2}_{ECSA} for 1T'-WS_2 NSs sample and 1.09 μA cm^{-2}_{ECSA} for 2H-WS_2 NSs sample. These results highlight the electrochemical activity of this WS_2 MSLs.

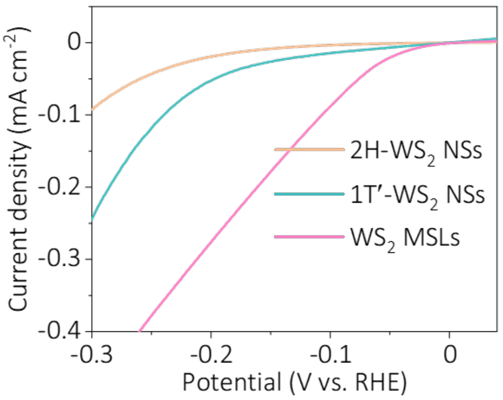

Supplementary Figure 25 | HER activity normalized for the electrochemical active surface area (ECSA).

To further demonstrate the enhanced intrinsic activity of WS_2 MSLs, the polarization curves were normalized to electrochemically active surface area (ECSA), which was derived from the double-layer capacitance (C_{dl}, **Supplementary Fig. 17–20**). As shown in **Supplementary Fig. 25** and **Supplementary Table 3**, WS_2 MSLs still reveals substantially higher HER current density than that of 1T'-WS_2 NSs and 2H-WS_2 NSs at the same overpotential under the same measurement conditions. It is important to emphasize that any use of J_{ECSA} values calculated from ECSA should be used as an approximate guide for comparing specific activity and should not be interpreted as an absolute reflection of turnover frequency, especially when comparing high-surface area and/or nanoporous films[2].

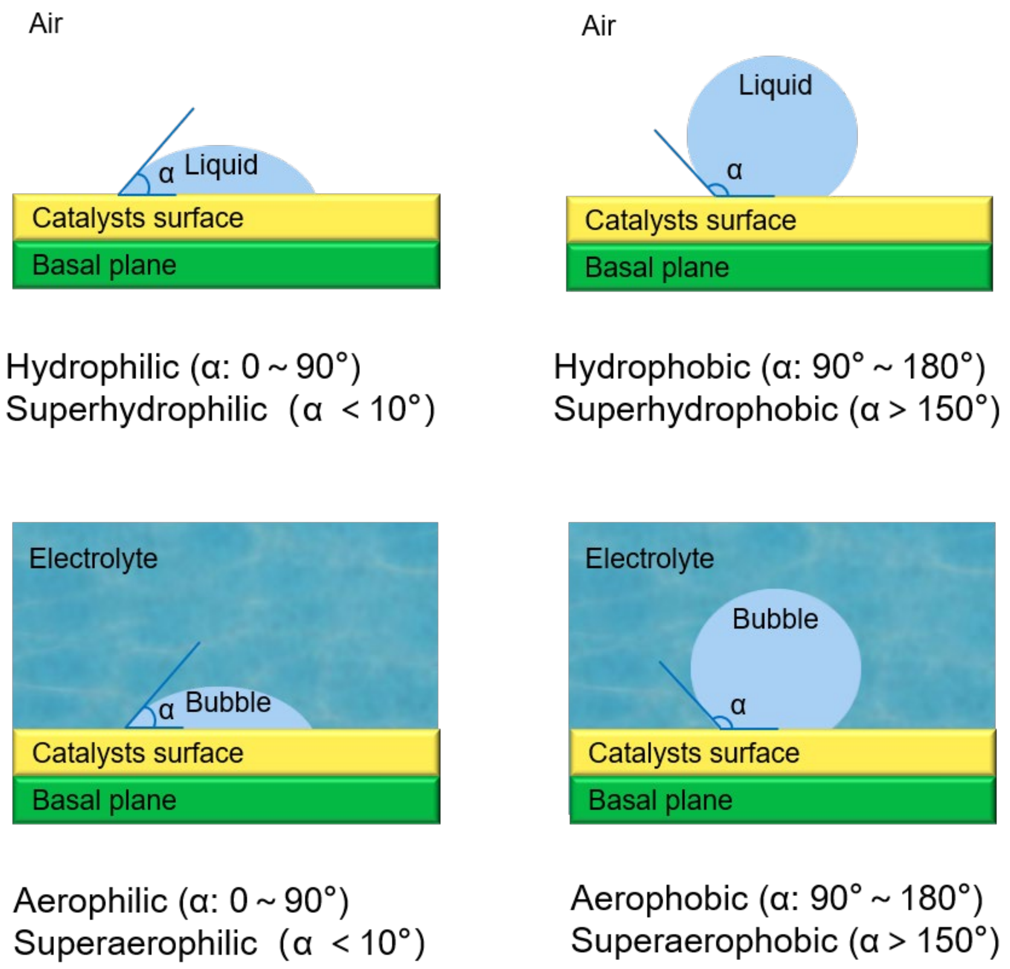

Supplementary Figure 26 | Schematic of the different wetting states that are possible on catalytic surfaces.

The terms "superaerophobicity" and "superaerophilicity" are analogous to the terms like "superhydrophobicity" and "superhydrophilicity", which are frequently used to describe the interaction between solid surface and water. Herein, since the investigated objects are moved from "liquid droplets" to "gas bubbles", the "hydro-" term is replaced by "aero-" and the investigation environments are changed from air to water[3]. Generally, a superhydrophobic surface shows a high liquid contact angle (LCA, >150°) and a tiny adhesion force, while a superhydrophilic surface is a surface with low LCA

(e.g., <10°) and a high adhesion force[4]. Thereby the superaerophobic surface is defined as a surface with high bubble contact angle (BCA, usually $\alpha > 150°$) underwater, which also shows a low adhesion force, while the superaerophilic surface means low BCA (usually <10°) and high adhesion force[5,6].

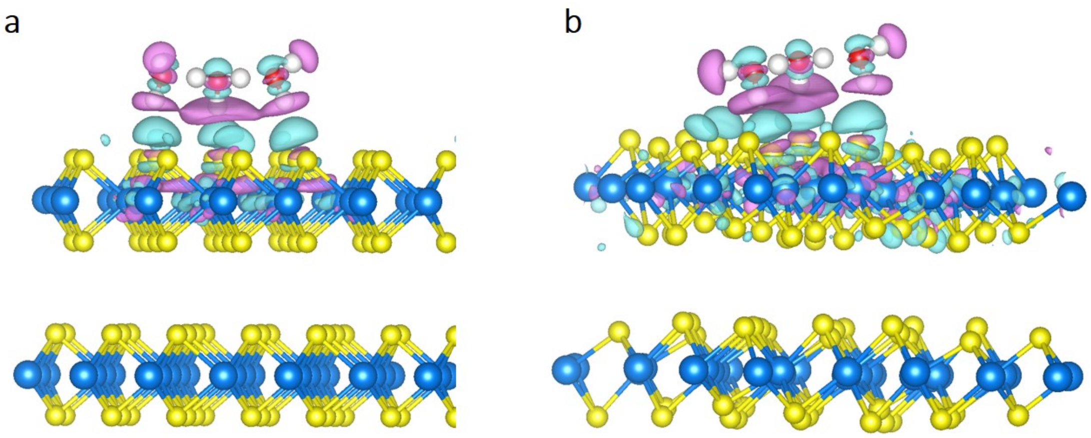

Supplementary Figure 27 |. The adsorption of hydrated cation onto the surface of 2H-WS_2 MSL and 1T'-WS_2 MSL, respectively.

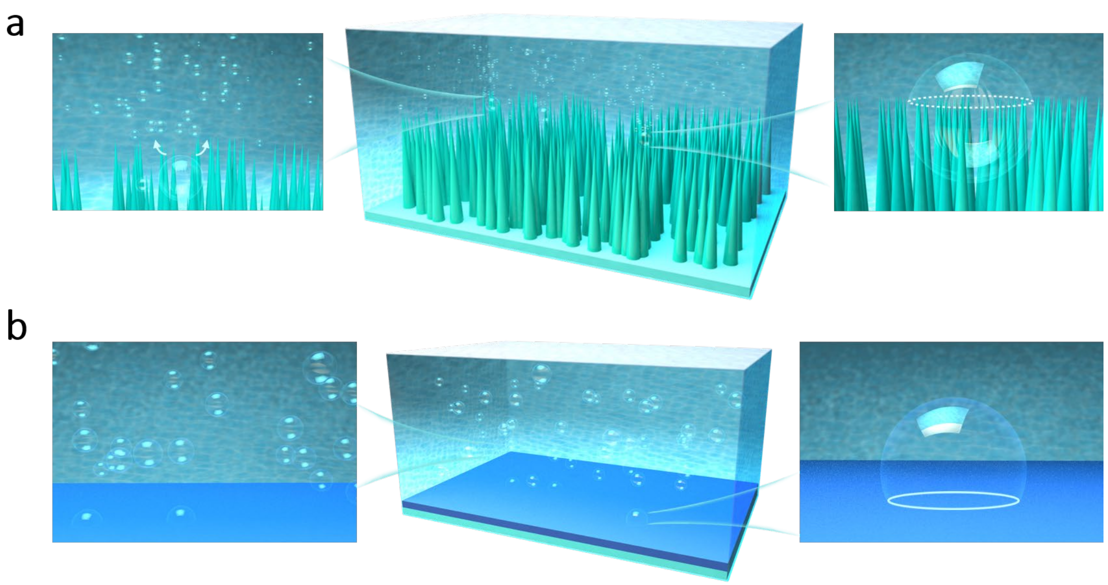

Supplementary Figure 28 | Schematic illustration of how the flat surface morphology affecting the bubble contacts and release.

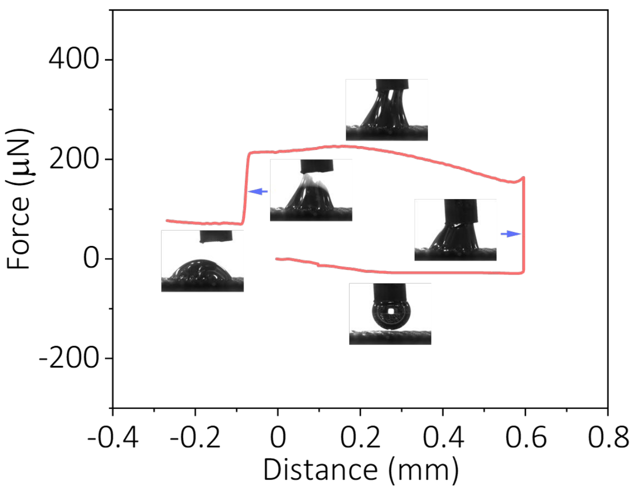

Supplementary Figure 29 | Adhesive forces measurements of the gas bubbles on bare CFC surface.

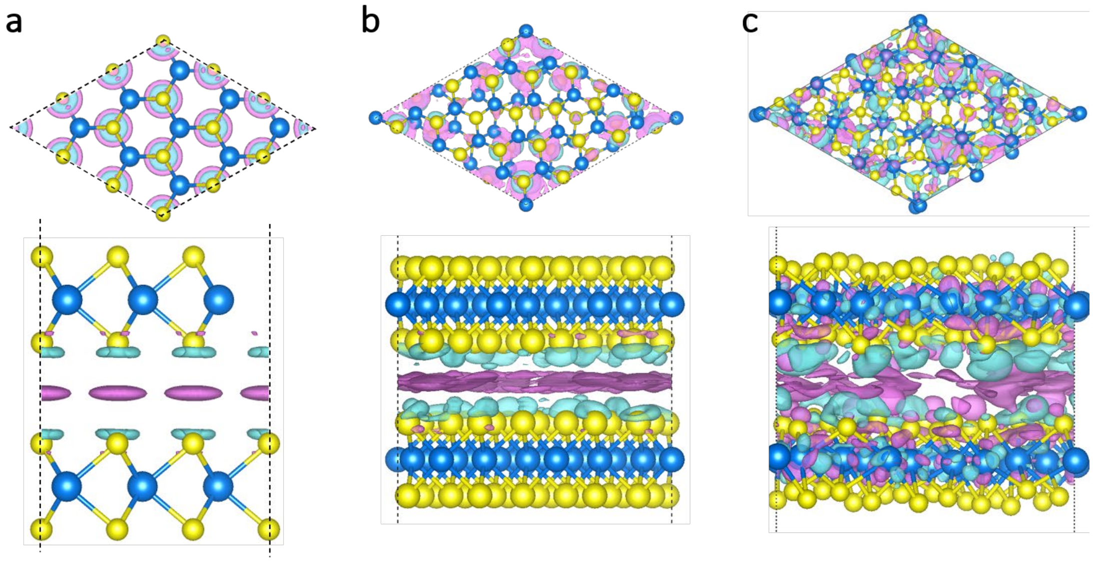

Supplementary Figure 30 | Spatial map of charge density difference for the twisted bilayer WS_2 (calculated as $\rho_{\mathrm{diff}} = \rho_{\mathrm{BL}} - \rho_{\mathrm{TOP}} - \rho_{\mathrm{BOT}}$). Regions of electron accumulation and depletion are denoted by red and blue lobes, respectively. Isosurface $\rho = 2 \times 10^{-4}$ e/bohr.

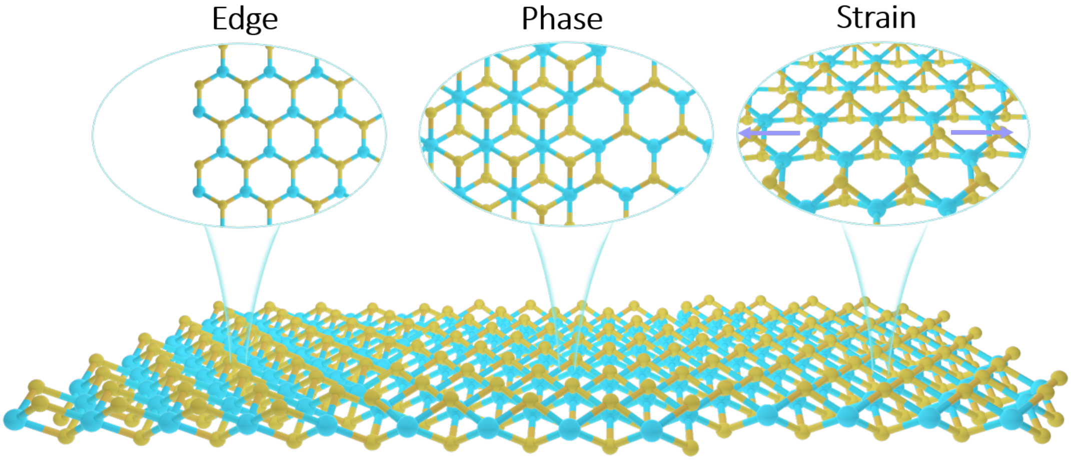

Supplementary Figure 31 | Various active sites of WS_2 for catalytic HERs. Yellow and cyan balls represent S and W atoms, respectively.

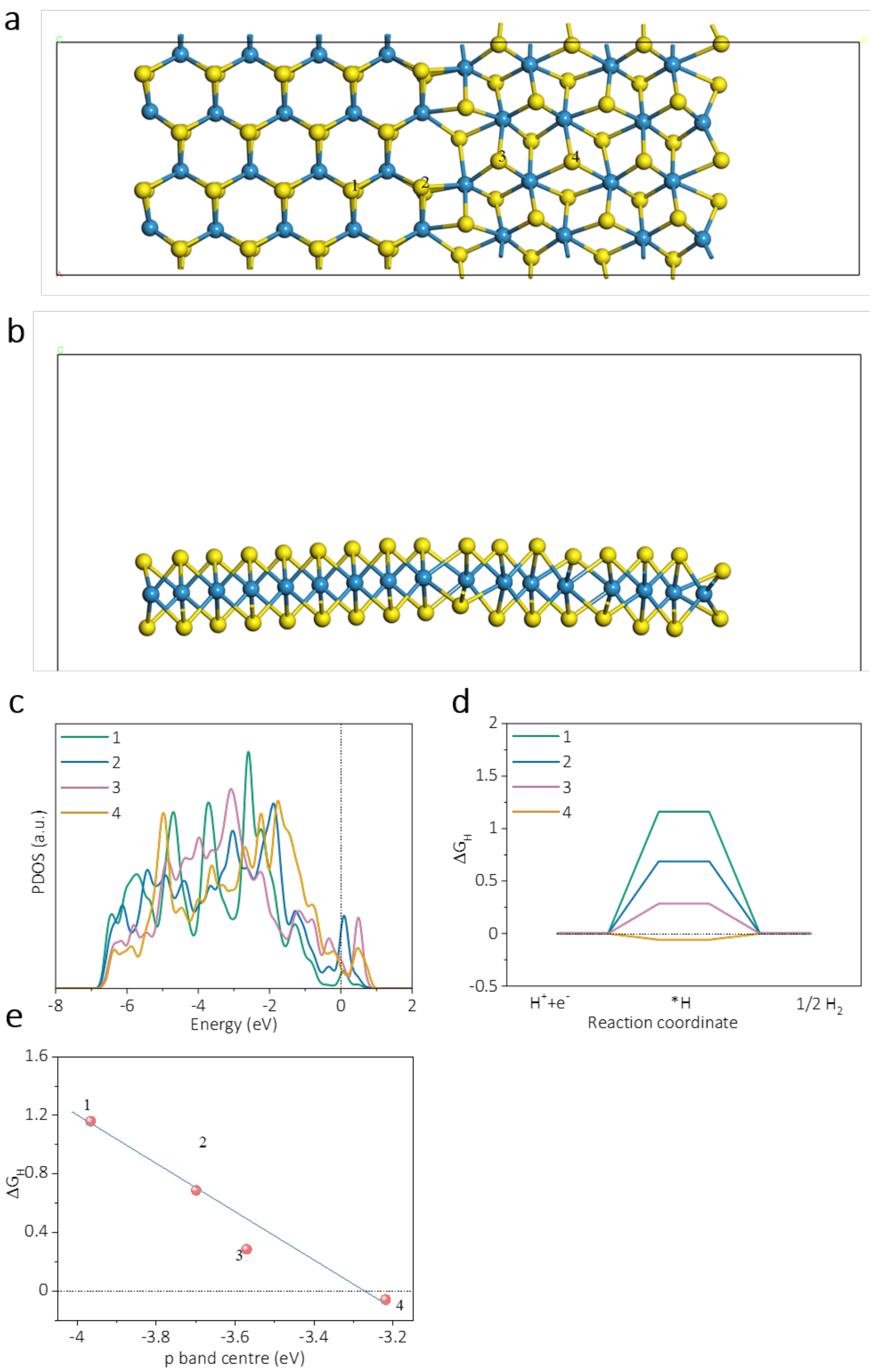

Supplementary Figure 32 | (a, b) Top and side perspective views (left to right) of the DFT calculated geometries for 1T′@2H heterojunctions of WS_2 nanobelts. (**c**) The p-projected density of states on the S atom binding sites. (**d**) ΔG_H of active sits corresponding to (a). (**e**) A linear relation between free energy and p band center.

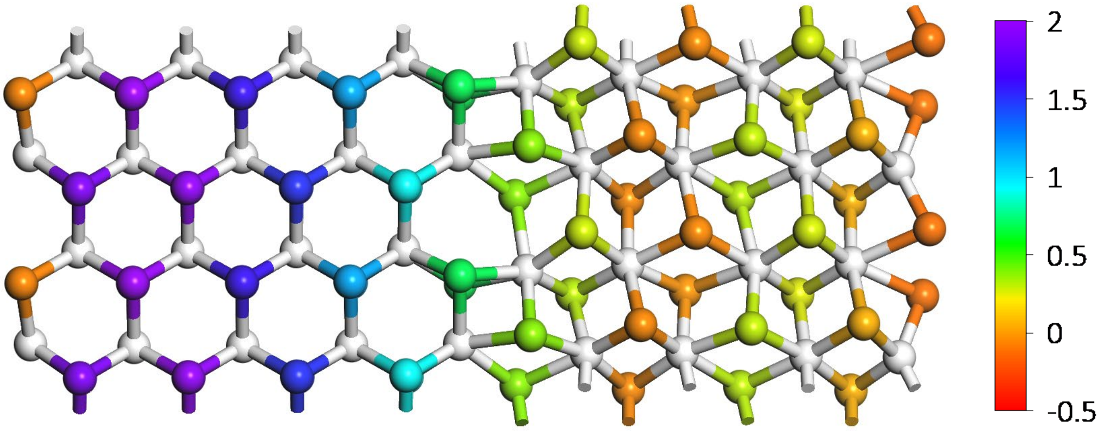

Supplementary Figure 33 | ΔG_H of all S sits balls. Colored balls represent S atom with their corresponding ΔG_H value and the white balls represent W atoms.

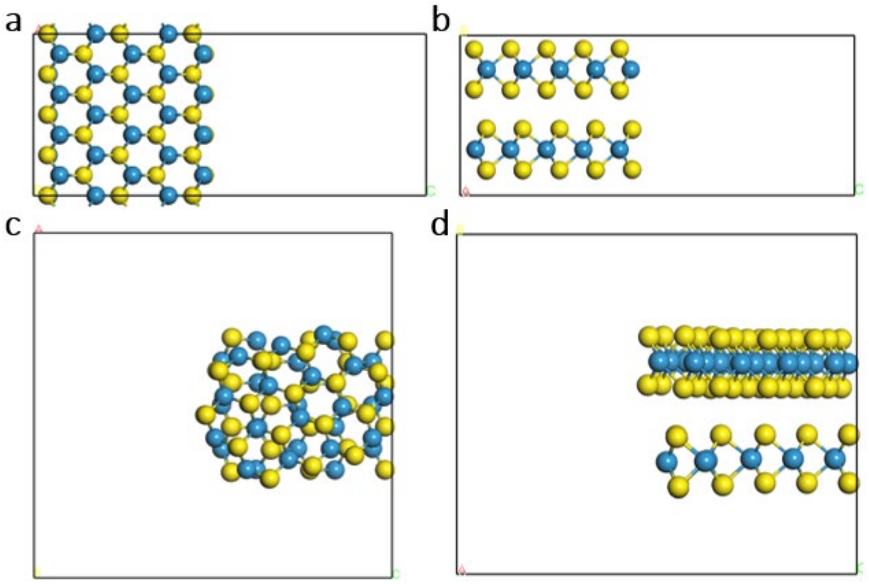

Supplementary Figure 34 | Top and side perspective views (left to right) of the DFT calculated geometries for bilayers 2H-WS_2 (**a**, **b**) and twisted bilayers WS_2 (**c**, **d**).

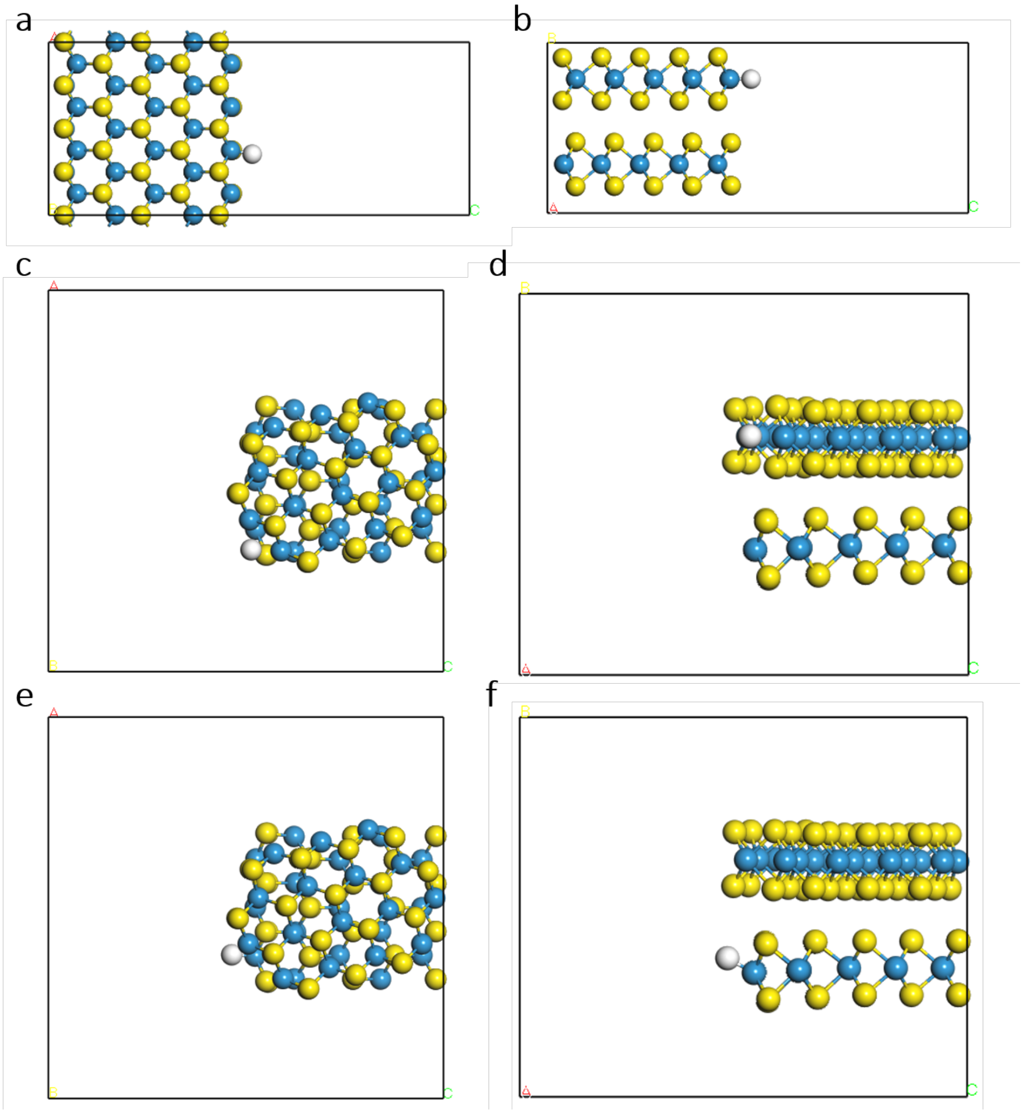

Supplementary Figure 35 | Top and side perspective views (left to right) of the DFT calculated geometries for H adsorbed at different active W sites of bilayers 2H-WS_2 (**a, b**) and twisted bilayers WS_2 (**c-f**).

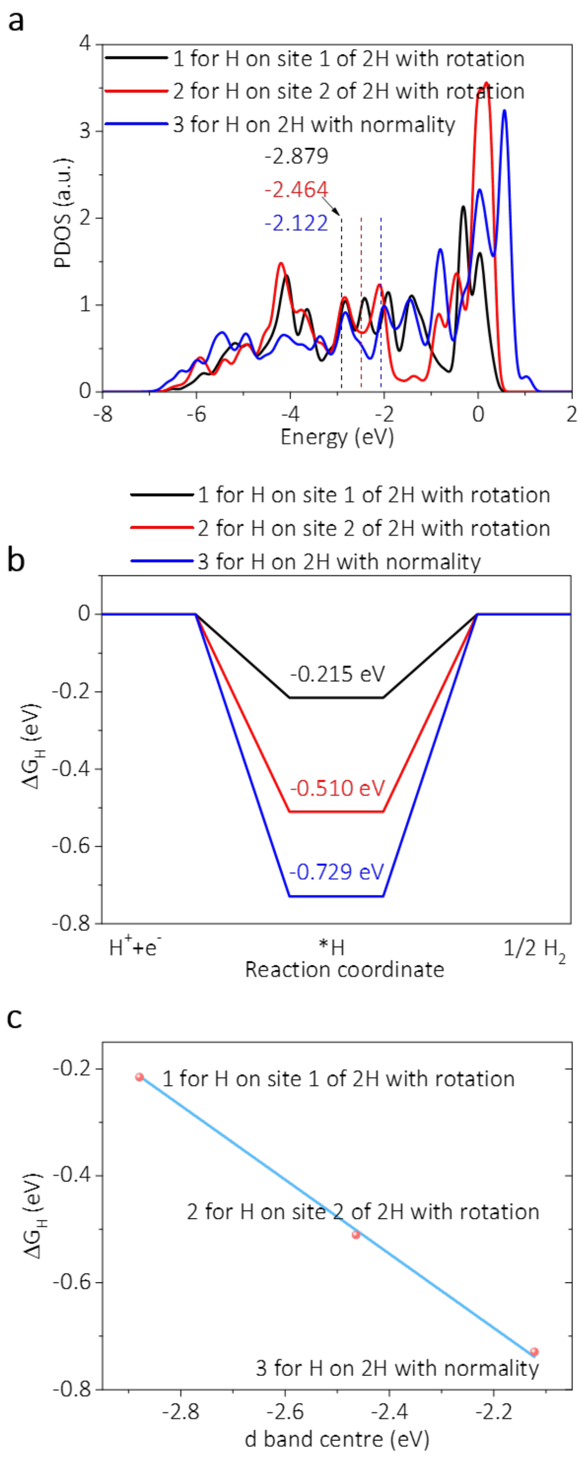

Supplementary Figure 36 | (a) The d-projected density of states on the W atom binding sites. **(b)** ΔG_H of active sits corresponding to the Supplementary Figure 13. **(c)** A linear relation between free energy and d band center.

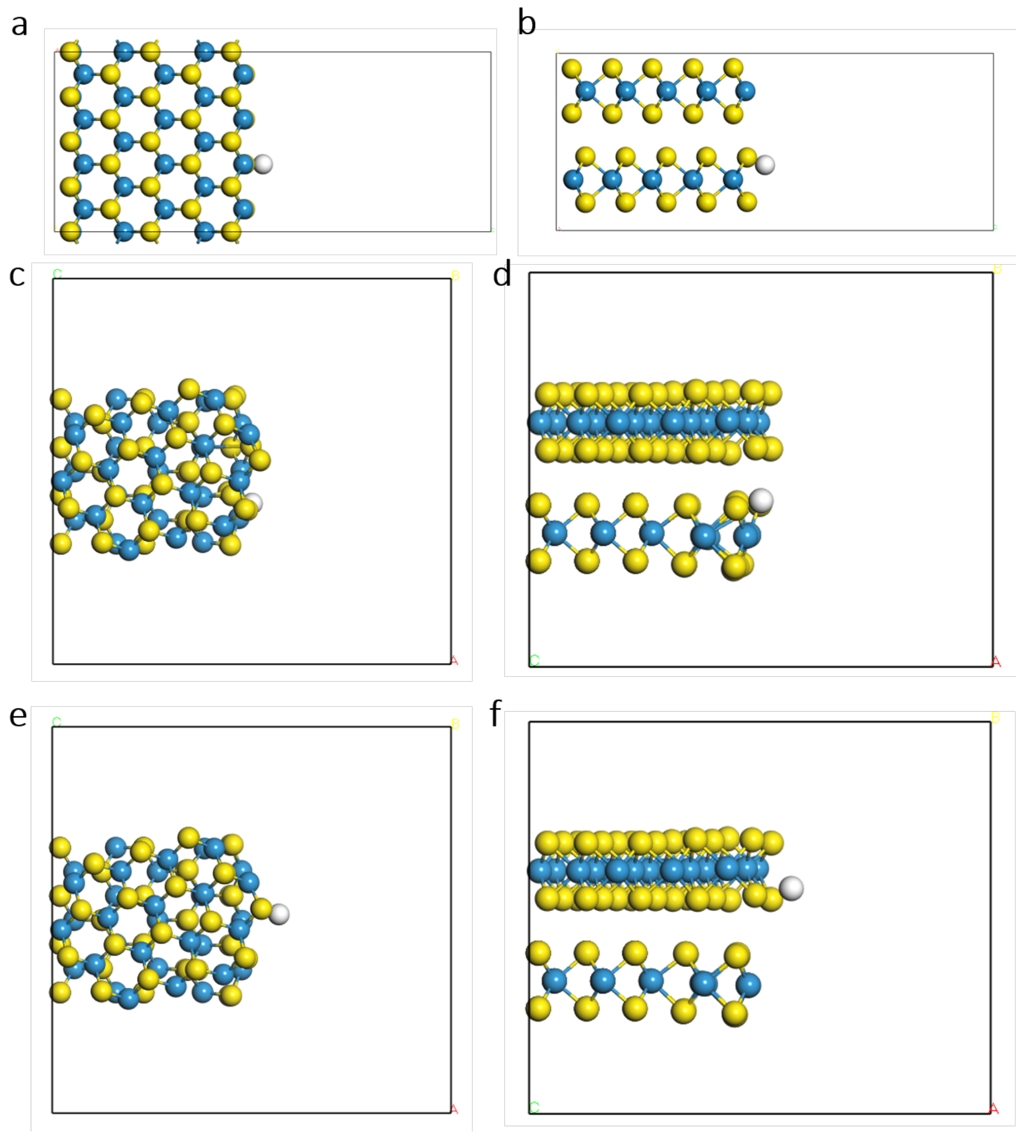

Supplementary Figure 37 | Top and side perspective views (left to right) of the DFT calculated geometries for H adsorbed at different active S sites of bilayers 2H-WS_2 (**a,** **b**) and twisted bilayers WS_2 (**c-f**).

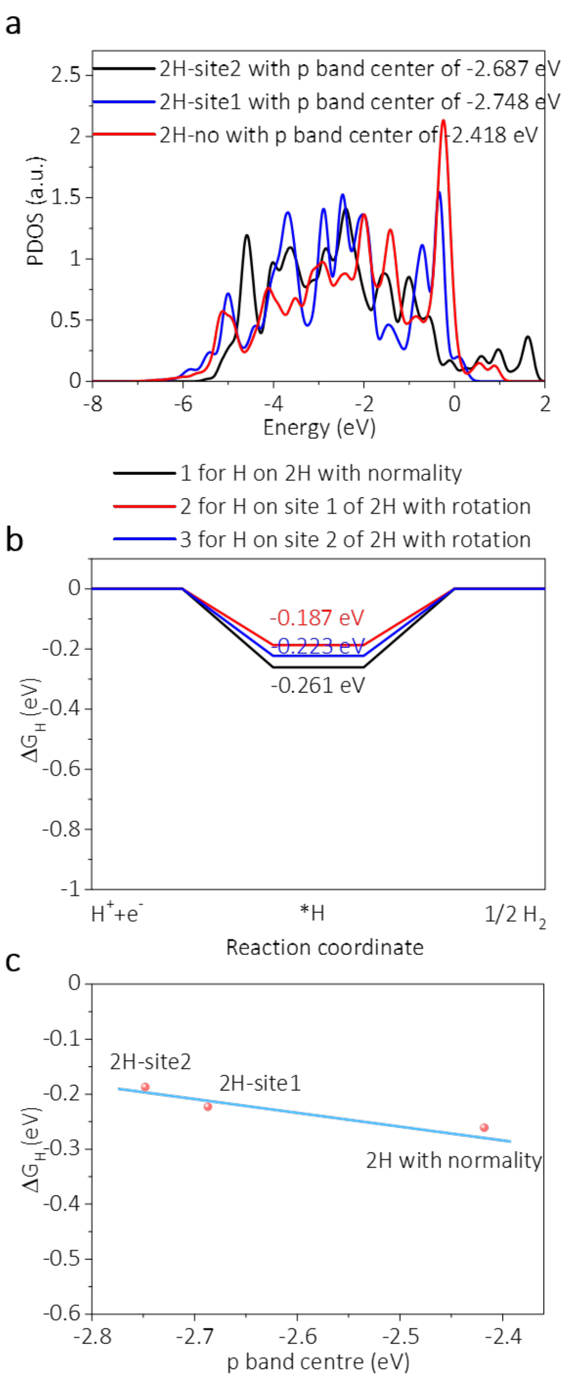

Supplementary Figure 38 | (a) The P-projected density of states on the S atom binding sites. (**b**) ΔG_H of active sits corresponding to the Supplementary Figure 15. (**c**) A linear relation between free energy and p band center.

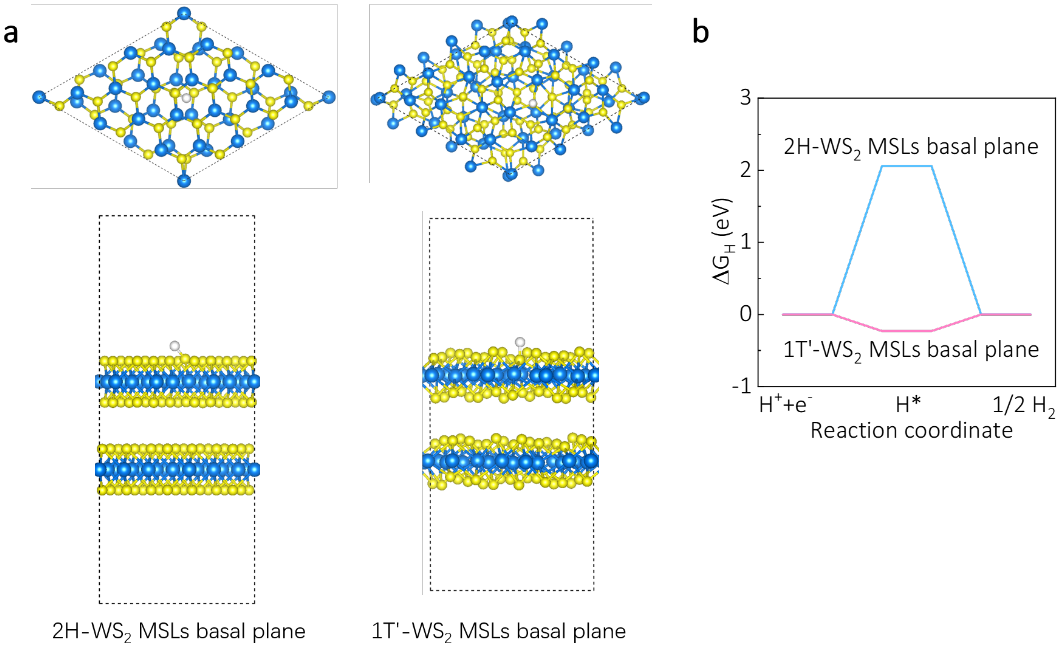

Supplementary Figure 39 | (**a**) Atomic models of hydrogen absorbed at the at the basal plane of 2H-WS_2 MSLs and 1T'-WS_2 MSLs, (**b**) Calculated Gibbs free energy (ΔG_H) diagram for hydrogen binding at the basal plane of 2H-WS_2 MSLs basal plane and 1T'-WS_2 MSLs.

Supplementary Table 1 | Summary of HER performance using WS_2 nanostructures as electrocatalysts.

Catalyst	Overpotential at *J* = 10 mA/cm^2 (mV)	Tafel Slope (mV/decade)	Stability Time/CV cycles	Reference
WS_2 MSLs	60	40	20h	*This work*
WS_2 nanodots	209	63	-	*Adv. Mater.* **2018**, *30*, 1705509.
WS_2 nanosheets	310	95	-	
$WS_{2(1-x)}P_{2x}$ nanoribbons	98	71	1000 cycles	*Small* **2017**, *13*, 1603706.
3D WS_2@P, N, O doped-graphene nanocomposites	125	52.7	20 h	*Adv. Mater.* **2015**, *27*, 4234.
WS_2 Quantum Dots	350	70	-	*Adv. Funct. Mater.* **2015**, *25*, 1127.
WS_2/WO_3 NHs	168	-	1000 cycles	*J. Mater. Chem. A* ***2019**, 7, 26378.*
WS_2@graphene	117	56	-	*Adv. Mater.* **2020**, *32*, 2002584.
1T'-D WS_2 nanosheets	200	50.4	12 h	*Nano Energy* ***2018**, 50, 176*
Exfoliated WS_2 nanosheets	234	55	-	*Nat. Mater.* ***2015**, 12, 850.*
WS_2 films	137	54	200 h	*J. Mater. Chem. A* ***2019**, 7, 22405.*

Supplementary Table 2 | Summary of some recently reported representative HER non-precious electrocatalysts.

Catalyst	Overpotential at *J* = 10 mA/cm^2 (mV)	Tafel Slope (mV/decade)	Reference
WS_2 MSLs	60	40	*This work*
MoS_2-60 s	131	48	*J. Am. Chem. Soc.* ***2020**, 142, 4298.*
Mo SAs/ML-MoS_2	170	40	*ACS Nano* ***2020**, 14, 767.*
MoS_2 NR	79	36.2	*Nat. Commun.* ***2020**, 11, 5032.*
Co-Pd-MoS_2	49.3	43.2	*Adv. Mater.* ***2020**, 32, e2001167.*
SV-MoS_2	170	60	*Nat. Mater.* ***2016**, 15, 48-53.*
mPF-Co-MoS_2	156	74	*Nat. Commun.* ***2017**, 5, 2995.*
Ni-CoP nanosheets	88	41	*Nano Lett.* ***2021**, 21, 823-832.*
Co-MoS_2 nanosheets	56	32	*Adv. Energy Mater.* ***2020**, 10, 2000291.*
Co-MoS_2	137	59	*Angew. Chem., Int. Ed.* ***2021**, 60, 7251-7258*
SA Co-D 1T MoS_2	42	32	*Nat. Commun.* ***2019**, 10, 5231.*
P_8W_{48}/rGO (phosphides)	28	38	*Energy Environ. Sci.* ***2016**, 9, 1012.*
Se-rich $MoSe_2$ nanosheets	130	46	*ACS Nano.* ***2020**, 14, 6295.*
N-MoS_2/CN	114	46.8	*J. Am. Chem. Soc.* ***2019**, 141, 18578.*
meso-Fe-MoS_2/$CoMo_2S_4$	142	90	*ACS Nano* ***2020**, 14, 4141.*

Supplementary Table 3 | Electrochemical values analysis of different studied WS_2 samples.

Samples	j_0 [μA cm_{ECSA}^{-2}]	Double-layer capacitance (C_{dl}) [mF cm^{-2}]	Electrochemical active surface area (ECSA) relative to WS_2 MSLs	Current density normalized to ECSA (J_{ECSA}) (η = −0.2 V) [μA cm^{-2}]
WS_2 MSLs	2.13	23.8	1	242.68
1T'-WS_2 NSs	1.55	15.2	0.64	32.75
2H-WS_2 NSs	1.09	11.4	0.48	24.14

Supplementary Table 4 | Impedance parameters for the equivalent circuit that was shown in Supplementary Figure 21.

Samples	R_s	CPE	R_{ct}
WS_2 MSLs	0.39	10.8	8.9
1T'-WS_2 NSs	0.17	15.1	10.5
2H-WS_2 NSs	0.10	27.3	57.6

Supplementary Table 5 | The TOF values of different WS_2 catalysts.

Catalyst	Potential (mV)	TOF (s^{-1})	Active site density (cm^{-2})
WS_2 MSLs	100	0.076–0.231	$(4.05–12.16) \times 10^{17}$
	150	0.159–0.477	
	200	0.246–0.739	
1T'-WS_2 NSs	100	0.007–0.022	$(2.74–8.26) \times 10^{17}$
	150	0.015–0.045	
	200	0.030–0.090	
2H-WS_2 NSs	100	0.004–0.014	$(1.81–5.47) \times 10^{17}$
	150	0.011–0.033	
	200	0.026–0.078	

Supplementary Table 6 | Comparison of mass activity of various WS_2-based electrocatalysts.

Catalysts	Mass activity (A g^{-1})	Reference
WS_2 MSLs	843.5 A g^{-1} at −300 mV	*This work*
1T'-WS_2 NSs	192.3 A g^{-1} at −300 mV	
2H-WS_2 NSs	109.6 A g^{-1} at −300 mV	
As-exfoliated WS_2 nanosheets	~92.8 A g^{-1} at −300 mV	*Nat. Mater.* ***2013****, 12, 850–855.*
1T'-D WS_2	~135.7 A g^{-1} at −300 mV	*Nano Energy,* ***2018,*** *50, 176–181.*
2H-WS_2	~44.46 A g^{-1} at −300 mV	
Compared WS_2	~387.3 at −250 mV	*Adv. Mater.* ***2018****, 30, 1705509*
WS_2 composite (DMF)	~141.6 A g^{-1} at −300 mV	*Adv. Funct. Mater.* ***2015****, 25, 1127–1136*

Supplementary Note 1

Electrochemical Active Surface Area (ECSA) Calculations

(1) Cyclic voltammograms (CV) method

Cyclic voltammetry (CV) was conducted to evaluate the electrochemical double layer capacitance (C_{dl}) of the materials at non-faradaic potentials as the means of estimating the corresponding electrochemical active surface areas. For CV measurements, a series of CV curves were performed at various scan rates (20, 40, 60, 80, 100, 120, 140 mV s^{-1}) in 0.25–0.30 V vs. RHE region. The cathodic and anodic charging currents measured at 0.275 V vs RHE plotted as a function of scan rate. The determined double-layer capacitance of the system is taken as the average of the absolute value of the slope of the linear fits to the data (**Supplementary Figure 17-20**). The specific capacitance values of WS_2 MSLs, 1T'-WS_2 NSs and 2H-WS_2 NSs are 23.8 mF cm^{-2}, 15.2 mF cm^{-2}, 11.4 mF cm^{-2} respectively. The background capacitance of the bare CFC electrode was subtracted from the obtained double layer capacitance to compensate for the low substrate coverage (**Supplementary Figure 17**).

(2) Electrochemical impedance spectroscopy (EIS) method

The double-layer capacitance was also measured using EIS in the same non-Faradaic region. A sinusoidal potential is applied to the system and the frequency-dependent complex impedance is measured. The example Nyquist plots of the real and imaginary components of the electrochemical impedance in a non-Faradaic region measured between 0.1 Hz and 100 kHz for different samples catalyst were shown in

Supplementary Figure 21. In the limit of high frequency and under non-Faradaic conditions, the electrochemical system is approximated by the modified Randles circuit shown in the inset of **Supplementary Figure 21**, where R_s is the solution resistance, CPE is a constant-phase element related to the double-layer capacitance, and R_{ct} is the charge-transfer resistance from any residual Faradaic processes. The simulation of the EIS spectra using an equivalent circuit model allowed us to determine the above three parameters. The determined double-layer capacitance of the WS_2 MSLs, 1T'-WS_2 NSs, and 2H-WS_2 NSs from the fitted data is 27.3 mF cm^{-2}, 15.1 mF cm^{-2}, 10.8 mF cm^{-2}, respectively (**Supplementary Table 4**). The double-layer capacitance we measured by EIS is within 15% of that measured from the scan rate-dependent CVs[7]

In order to confirm the determination of the electrochemically active surface area (ECSA) of the catalysts, the following equation[2,7–9] could be utilized below:

$$A_{ECSA} = \frac{C_{dl}}{C_{specific}}$$

where C_{dl} is double-layer capacitance, $C_{specific}$ is specific capacitance.

In general, the ECSA estimates to be accurate within about an order of magnitude, and emphasize that the values should be considered only as an approximate guide for comparing electroactive surface area. The specific capacitance is converted into an electrochemical active surface area (ECSA) using the specific capacitance value for a flat standard with 1 cm^2 of real surface area. The specific capacitance for a flat surface is generally found to be in the range of 20–60 μF cm^{-2}. In the following calculations of

ECSA, we assume the value of 60 μF cm^{-2} as the specific capacitance of the catalysts in this work[2,9–12]. Based on the above equation analysis, the detailed calculation process is as follows:

Calculated electrochemical active surface area (ECSA):

(**CV method**) I: WS_2 MSLs II: 1T'-WS_2 NSs III: 2H-WS_2 NSs

$$A^{I}_{ECSA} = \frac{23.8\ \text{mF cm}^{-2}}{60\ \mu\text{m cm}^{-2}\ \text{per cm}^{2}_{\text{ECSA}}} = 396.6\ \text{cm}^{2}_{\text{ECSA}}$$

$$A^{II}_{ECSA} = \frac{15.2\ \text{mF cm}^{-2}}{60\ \mu\text{m cm}^{-2}\ \text{per cm}^{2}_{\text{ECSA}}} = 253.3\ \text{cm}^{2}_{\text{ECSA}}$$

$$A^{III}_{ECSA} = \frac{11.4\ \text{mF cm}^{-2}}{60\ \mu\text{m cm}^{-2}\ \text{per cm}^{2}_{\text{ECSA}}} = 190.0\ \text{cm}^{2}_{\text{ECSA}}$$

Using the same calculation method, the electrochemical active surface area (ECSA) of WS_2 MSLs, 1T'-WS_2 NSs and 2H-WS_2 NSs determined by EIS method are 455.0 cm^{2}_{ECSA}, 251.6 cm^{2}_{ECSA}, 180.0 cm^{2}_{ECSA}, respectively.

Supplementary Note 2

Calculation of turn over frequency (TOF)

To calculate the active surface site density and per-site TOF for the WS_2 catalyst, we adopt the method applied by Jaramillo et al.[13,14], where the relative roughness factor (RF) of the catalyst, the geometry of WS_2 surface, and the HER current density are used. As shown in **Supplementary Figure 18**, we have determined the specific capacitance to be 23.8 mF cm^{-2}, which can be directly used to estimate the relevant electrochemical active surface area (ECSA) by using the specific capacitance value for a flat electrode with real surface area 1 cm^2. We assume 60 μF cm^{-2} for a flat electrode provided in Jaramillo et al[13,15] for calculation here, and use 20 and 60 μF cm^{-2} for evaluating a lower and upper limit of the TOFs[8,16] (**Supplementary Table 5**).

The number of electrochemically accessible surface sites on the WS_2 MSLs catalyst can be calculated by using the following formula:

$$\frac{\#\ Surface\ sites\ (catalyst)}{cm^2\ geometric\ area} = \frac{\#\ Surface\ sites\ (flat\ standard)}{cm^2\ geometric\ area} \times Roughness\ factor$$

Compared to the flat standard electrode (60 μF cm^{-2}), the relative roughness factor of the investigated catalyst is determined to be ~396.6 based on the electrochemically double-layer capacitance measurement. As a result, the number of surface active sites for the WS_2 MSLs catalyst is estimated to be 12.16×10^{17} surface sites cm^{-2} from the above formula, indicating a large number of active sites introduced by our special experimental design.

To further get insights into the per-site TOF, the following formula is utilized:

$$TOF\ per\ site = \frac{\#\ Total\ Hydrogen\ Turn\ Over\ /\ cm^2\ geometric\ area}{\#\ Surface\ Sites\ (Catalyst)\ /\ cm^2\ geometric\ area}$$

In this formula, the total number of hydrogen turn over events per geometric area at 1 mA cm^{-2} is close to $3.12 \times 10^{15}\ \frac{\mathrm{H_2\ /\ s}}{\mathrm{cm^2}}$ according to Jaramillo *et al.*'s work[13,17,18]. So the TOF per site for our investigated hybrid catalyst at different overpotentials vs. RHE and pH = 0 is calculated as follows:

At η = 100 mV, $\left(3.12 \times 10^{15}\ \frac{H_2\ /\ s}{cm^2} / \frac{mA}{cm^2}\right)\left(30\ \frac{mA}{cm^2}\right)\left(\frac{1\ cm^2}{6.0 \times 10^{17}\ surface\ sites}\right) = 0.156\ \frac{H_2\ /\ s}{surface\ site}$

At η = 150 mV, $\left(3.12 \times 10^{15}\ \frac{H_2\ /\ s}{cm^2} / \frac{mA}{cm^2}\right)\left(62\ \frac{mA}{cm^2}\right)\left(\frac{1\ cm^2}{6.0 \times 10^{17}\ surface\ sites}\right) = 0.322\ \frac{H_2\ /\ s}{surface\ site}$

At η = 200 mV, $\left(3.12 \times 10^{15}\ \frac{H_2\ /\ s}{cm^2} / \frac{mA}{cm^2}\right)\left(96\ \frac{mA}{cm^2}\right)\left(\frac{1\ cm^2}{6.0 \times 10^{17}\ surface\ sites}\right) = 0.499\ \frac{H_2\ /\ s}{surface\ site}$

We use 20 and 60 μF cm^{-2} for evaluating a lower and upper limit of the TOFs of all samples (capacitance method)[13], and the obtained TOF range values are listed in **Supplementary Table 5**.

Supplementary Note 3

Calculated mass activity

Mass activity (A g^{-1}) is derived from the current density that normalized by the mass loading (0.196 mg cm^{-2}) at a certain overpotential. The mass activity of WS_2 MSLs can be calculated by the following equation:

$$Mass\ activity = \frac{J}{M}$$

Where J is current density, M is mass loading. For instance, the current density for WS_2 MSLs is 168.7 mA cm^{-2} at the overpotential of 300 mV, the mass activity of WS_2 MSLs is calculated to be 843.5 A g^{-1}.

Supplementary Note 4

DFT calculations

We perform density functional theory (DFT) calculations using the plane wave code Vienna Ab-initio Simulation Package (VASP)[19], 31 with supplied projector augmented wave (PAW) potentials 32 for W (including 4p electrons as valence) and S. To treat the exchange-correlation, we use the generalized gradient approximation (GGA) of Perdew-Becke-Ernzerhof (PBE)[20]. Van der Waals (vdW) interactions are included with the DFT-D2 method of Grimme[21]. An energy cutoff of 750 eV was used and a k-point sampling set of 3 × 3 × 1 were tested to be converged. A force tolerance of 0.01 eV $Å^{-1}$, energy tolerance of 5.0×10^{-7} eV per atom and maximum displacement of 5.0×10^{-4} Å were considered. The Grimme method for DFT-D correction is considered for all calculations[22]. Each atom in the storage models is allowed to relax to the minimum in the enthalpy without any constraints to calculate the surface energy. In order to test the HER activity on the edge sites of WS_2 MSLs, we modeled a nanoribbon structure with a vacuum of 15 Å in the y-direction. Two kinds of edge structures are created by the above procedure, including the one that ends with the exposed W atoms and the other ending with S only.

The Hydrogen Adsorption Free Energy: ΔG_H, is determined in the same way as in previous studies. The adsorption energy is defined as

$$\Delta E_H = E(WS_2+H) - E(WS_2) - ½\, E(H_2) \qquad (1)$$

where (WS_2+H) refers to hydrogen adsorbed on the WS_2 surface, (WS_2) refers to a

clean WS_2 surface, and H_2 refers to gas phase hydrogen molecule. The hydrogen adsorption free energy is calculated at zero potential and pH = 0 as

$$\Delta G_H = \Delta E_H + \Delta E_{zep} - T\Delta S \qquad (2)$$

where ΔE_H is the hydrogen adsorption energy, ΔE_{zep} is the difference in zero point energy, T is the temperature (300 K) and ΔS is the difference in entropy between H that is adsorbed and in the gas phase at 101325 Pa. A normal mode analysis is used to determine the vibrational frequencies of the adsorbed species, which are used to determine the zero-point energy correction and the entropy. The adsorption is too strong if ΔG_H is very negative or too weak if ΔG_H is very positive.

Supplementary Note 5

Finite-element calculation

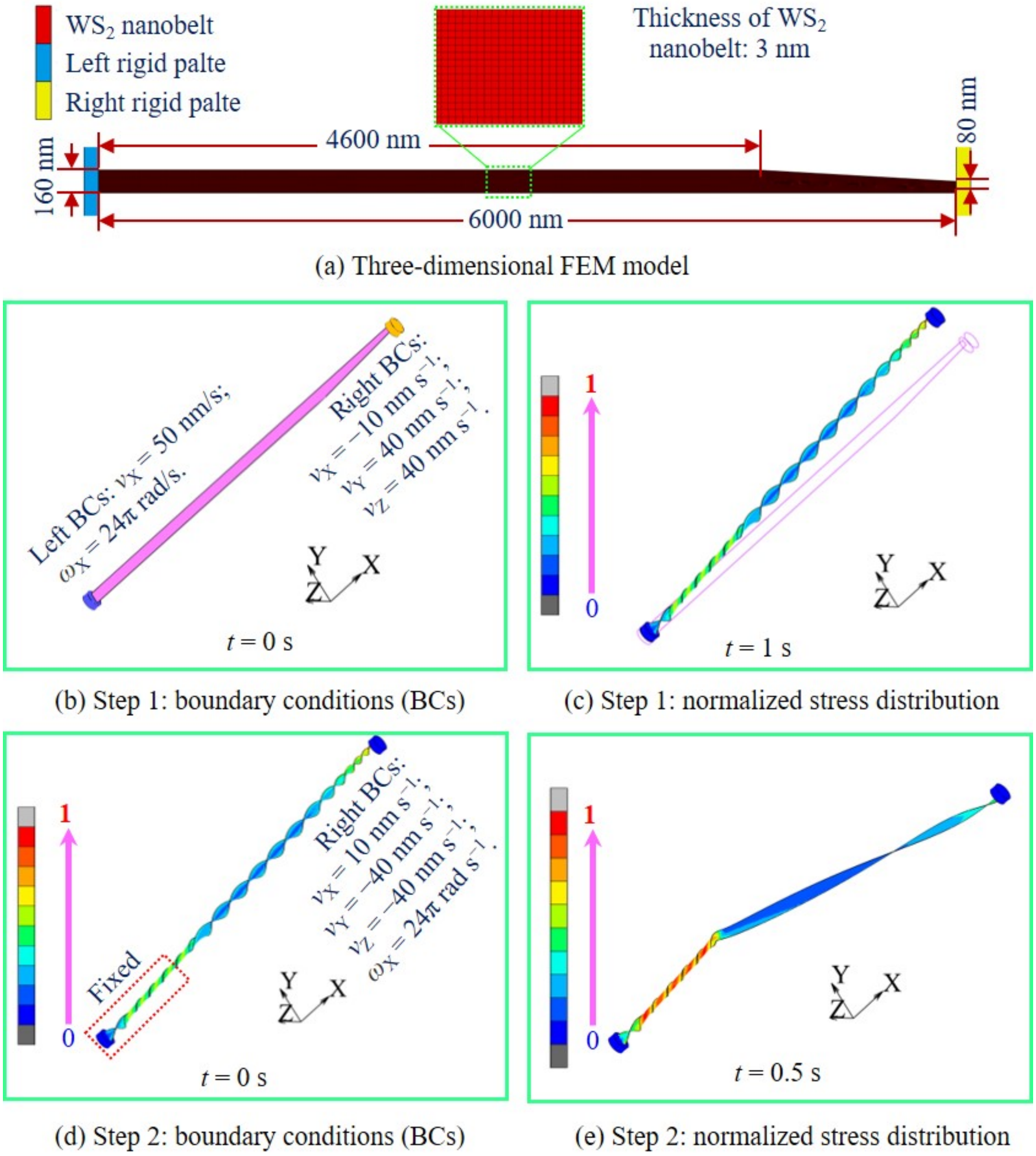

For a mechanical perspective, the formation of a WS_2 nanocone under unbalanced external forces could be deemed as the WS_2 nanobelt's screwing process, which can be numerically modeled by using the typically finite element method (FEM). The above table (a) shows the designed three-dimensional FEM model, wherein the WS_2 nanobelt

is set as an elastic body meshed with 57045 eight-node hexahedral elements. The density, Young's modulus, and Poisson's ratio of the WS_2 nanobelt are specified as 7.51 g cm^{-3}, 272 GPa, and 0.22, respectively[23]. Two steps (screwing and partial release) are specified to approximate the WS_2 nanobelt's screwing process, wherein the calculated results (such as stress and strain distributions) in Step 1 are taken as the pre-state for Step 2. The above table (b) and (d) specify, respectively, the mechanical boundary conditions (BCs) in Steps 1 and 2, and the above table (c) and (e) show the calculated results (normalized stress distributions) of the screwed WS_2 nanobelt, respectively.

Supplementary References

1. Kibsgaard J, Jaramillo TF, Besenbacher F. Building an appropriate active-site motif into a hydrogen-evolution catalyst with thiomolybdate $[Mo_3S_{13}]^{2-}$ clusters. *Nat. Chem.* **6**, 248–253 (2014).
2. McCrory CC, Jung S, Ferrer IM, Chatman SM, Peters JC, Jaramillo TF. Benchmarking hydrogen evolving reaction and oxygen evolving reaction electrocatalysts for solar water splitting devices. *J. Am. Chem. Soc.* **137**, 4347–4357 (2015).
3. Wang S, Liu K, Yao X, Jiang L. Bioinspired surfaces with superwettability: new insight on theory, design, and applications. *Chem. Rev.* **115**, 8230–8293 (2015).
4. Lu Z, et al. Ultrahigh hydrogen evolution performance of under-water "superaerophobic" MoS_2 nanostructured electrodes. *Adv. Mater.* **26**, 2683–2687, 2615 (2014).
5. Liu M, Wang S, Jiang L. Nature-inspired superwettability systems. *Nat. Rev. Mater.* **2**, 17036 (2017).
6. Xu W, Lu Z, Sun X, Jiang L, Duan X. Superwetting electrodes for gas-involving electrocatalysis. *Acc. Chem. Res.* **51**, 1590–1598 (2018).
7. McCrory CC, Jung S, Peters JC, Jaramillo TF. Benchmarking heterogeneous electrocatalysts for the oxygen evolution reaction. *J. Am. Chem. Soc.* **135**, 16977–16987 (2013).
8. Kibsgaard J, Jaramillo TF. Molybdenum phosphosulfide: An active, acid-stable, earth-abundant catalyst for the hydrogen evolution reaction. *Angew. Chem. Int. Ed.* **53**, 14433–14437 (2014).
9. Kibsgaard J, et al. Designing an improved transition metal phosphide catalyst for hydrogen evolution using experimental and theoretical trends. *Energy Environ. Sci.* **8**, 3022–3029 (2015).
10. Gauthier JA, et al. Transition metal arsenide catalysts for the hydrogen evolution reaction. *J. Phys. Chem. C* **123**, 24007–24012 (2019).

11. Benck JD, Hellstern TR, Kibsgaard J, Chakthranont P, Jaramillo TF. Catalyzing the hydrogen evolution reaction (HER) with molybdenum sulfide nanomaterials. *ACS Catal.* **4**, 3957–3971 (2014).
12. Li H, et al. Amorphous nickel-cobalt complexes hybridized with 1T-phase molybdenum disulfide via hydrazine-induced phase transformation for water splitting. *Nat. Commun.* **8**, 15377 (2017).
13. Benck JD, Chen Z, Kuritzky LY, Forman AJ, Jaramillo TF. Amorphous molybdenum sulfide catalysts for electrochemical hydrogen production: insights into the origin of their catalytic activity. *ACS Catal.* **2,** 1916–1923 (2012).
14. Sanchez J, Hellstern TR, King LA, Jaramillo TF. Surface engineering of 3D gas diffusion electrodes for high-performance H_2 production with nonprecious metal catalysts. *Adv. Energy Mater.* **9**, 1901824 (2019).
15. Kibsgaard J, Chen Z, Reinecke BN, Jaramillo TF. Engineering the surface structure of MoS_2 to preferentially expose active edge sites for electrocatalysis. *Nat. Mater.* **11**, 963–969 (2012).
16. Li DJ, et al. Molybdenum sulfide/N-doped CNT forest hybrid catalysts for high-performance hydrogen evolution reaction. *Nano Lett.* **14**, 1228–1233 (2014).
17. Hellstern TR, Benck JD, Kibsgaard J, Hahn C, Jaramillo TF. Engineering cobalt phosphide (CoP) thin film catalysts for enhanced hydrogen evolution activity on silicon photocathodes. *Adv. Energy Mater.* **6**, 1501758 (2016).
18. Hellstern TR, et al. Investigating catalyst–support interactions to improve the hydrogen evolution reaction activity of thiomolybdate $[Mo_3S_{13}]^{2-}$ nanoclusters. *ACS Catal.* **7**, 7126–7130 (2017).
19. M. Segall, et al. First-principles simulation: ideas, illustrations and the CASTEP code, *J. Phys.: Condens. Matter* **14**, 2717–2744 (2002).
20. J.P. Perdew, K. Burke, M. Ernzerhof, Generalized gradient approximation made simple, *Phys. Rev. Lett.* **77**, 3865–3868 (1996).
21. D. Hamann, M. Schlüter, C. Chiang, Norm-conserving pseudopotentials, *Phys. Rev. Lett.* **43**, 1494–1497 (1979).

22. Grimme S. Semiempirical GGA-type density functional constructed with a long-range dispersion correction. *J. Comput. Chem.* **27**, 1787–1799 (2006).
23. Liu K, et al. Elastic properties of chemical-vapor-deposited monolayer MoS_2, WS_2, and their bilayer heterostructures. *Nano Lett.* **14**, 5097–5103 (2014).
