# Supplementary material for: WS2 moiré superlattices derived from mechanical flexibility for hydrogen evolution reaction
Source: Nat Commun. 2021 Aug 20;12:5070. doi: 10.1038/s41467-021-25381-1 (PMC8379161; doi:10.1038/s41467-021-25381-1)
Supplement: Supplementary file 3 — Description of Additional Supplementary Files [file 41467_2021_25381_MOESM3_ESM.docx]

**Description of Additional Supplementary Files**

**Supplementary Movie 1.** Desorption of bubbles on the ideal flat electrode surface.

**Supplementary Movie 2.** Desorption of bubbles on the conical nanoarrays surface.

**Supplementary Movie 3.** Adhesive forces measurements of the gas bubbles on WS2 MSLs-CFC surface.

**Supplementary Movie 4.** Adhesive forces measurements of the gas bubbles on bare CFC surface.
